# A community-codesigned LLM-powered chatbot for primary care: a randomized controlled trial

**DOI:** 10.1038/s44360-025-00021-w

**Published:** 2026-01-19

**Authors:** Sairan Li, Yanzeng Li, Shuya Zhou, Xinge Tao, Changjie Yu, Muzi Shen, Wangyue Chen, En Meng, Boyou Wu, Qirui Huang, Frances S. Mair, Jinchao Zhang, Jie Zhou, Lei Zou, Shasha Han

**Affiliations:** 1 School of Population Medicine and Public Health, Chinese Academy of Medical Sciences and Peking Union Medical College, Beijing, China; 2 Institute of Artificial Intelligence and Future Networks, Beijing Normal University, Zhuhai, China; 3 Wangxuan Institute of Computer Technology, Peking University, Beijing, China; 4 State Key Laboratory of General Artificial Intelligence, Peking University, Beijing, China; 5 National Research Institute for Family Planning, Chinese Academy of Medical Sciences and Peking Union Medical College, Beijing, China; 6 School of Science, Harbin Institute of Technology, Weihai, China; 7 School of Statistics, East China Normal University, Shanghai, China; 8 School of Health and Wellbeing, College of Medicine, Veterinary and Life Sciences, University of Glasgow, Glasgow, UK; 9 Pattern Recognition Center, WeChat AI, Tencent Inc., Beijing, China; 10 State Key Laboratory of Respiratory Health and Multimorbidity, Beijing, China; 11 Key Laboratory of Pathogen Infection Prevention and Control (Peking Union Medical College), Ministry of Education, Beijing, China

**Keywords:** Randomized controlled trials, Health services, Public health, Translational research

## Abstract

With a global shortage of primary healthcare physicians—particularly in resource-limited settings—large language models (LLMs) have the potential to support and enhance patients’ health awareness. Here we developed P&P Care (Population Medicine and Public Health), an LLM-powered primary care chatbot using a dual-track role-play codesign framework where community stakeholders and researchers simulated each another’s perspectives across four phases: contextual understanding; cocreation; testing and refinement; and implementation and evolution. The codesigned chatbot was integrated with e-learning modules and tested in a randomized controlled trial. The trial included 2,113 participants (1,052 women and 1,061 men) from urban and rural areas across 11 Chinese provinces who were randomly assigned to receive a consultation either with preparatory e-learning via the P&P Care or without. The study met its primary endpoint with the e-learning group showing significantly higher objective health awareness (mean score 2.95 ± 1.22) compared with the consultation-only group (mean score 2.34 ± 1.02; *P* < 0.001). Codesign offers a scalable solution for deploying LLMs in resource-limited settings. Chinese Clinical Trial Registry identifier: ChiCTR2500098101.

## Abstract (web summary)

A multimedia e-learning chatbot codesigned with community stakeholders increased health awareness in a randomized trial of 2,113 participants in urban and rural areas across 11 Chinese provinces.

## Main

Primary care is the cornerstone of a high-functioning health system, yet it faces a pressing workforce crisis worldwide^1^. This shortfall stems from a global deficit of medical trainees pursuing primary care careers and a lack of substantive community-based training, a gap evident across all health systems and exacerbated by structural deficits in low- and middle-income countries^2–6^. Global disparities are stark: between 2018 and 2022, Eastern Africa averaged 0.9 general practitioners per 10,000 people compared with 12.8 in Western Europe (data from the Population Reference Bureau); despite the Healthy China 2030 initiative, China’s rate remains low at 2.77 (ref. ^7^). Compounding this shortage, ageing populations and a rising burden of multimorbidity strain health systems globally^8,9^, demanding continuous, coordinated care that is increasingly beyond the reach of overwhelmed primary care^10–12^. This multifaceted crisis necessitates solutions that not only augment the workforce but also strengthen patient agency and health awareness.

Large language models (LLMs) that generate human-like text in response to user inquiries demonstrate emergent capabilities in clinical reasoning and empathetic communication^13–16^, offering a potential pathway to bridge this gap by empowering patient self-care. Their ability to provide personalized health guidance and chronic care management could be transformative, particularly in resource-limited settings. However, current LLM deployment remains confined to well-resourced hospitals^17–20^, by-passing the rural and resource-poor areas where the needs are greatest. This disparity highlights the urgent need for scalable, patient-facing LLM tools designed for underserved communities.

The effectiveness of such tools, however, hinges on a concept we term artificial intelligence (AI) health literacy—user proficiency in interacting with AI. Deficiencies in this literacy can lead to suboptimal interactions, wherein vague or overly generalized user input impedes the generation of clinically relevant responses^21^. Furthermore, inherent biases in training data and the potential for model hallucinations can produce plausible but inaccurate information, which demands critical evaluation skills^22^. Beyond user literacy, LLM deployment in underserved communities faces infrastructural hurdles, notably inadequate and prohibitively expensive network connectivity^23,24^, which stands in contrast to the infrastructure supporting hospital-based LLM deployment and presents a critical barrier to equitable access.

**Fig. 1:**
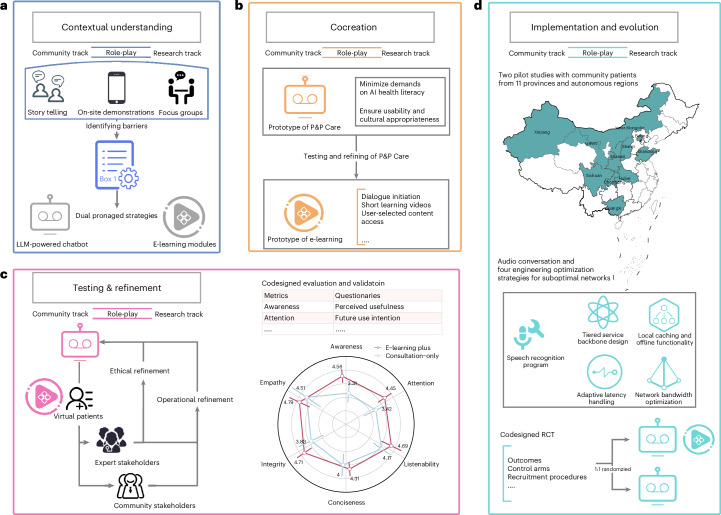
Dual-track role-play codesign process for developing, deploying and evaluating P&P Care. **a**, Contextual understanding: Community stakeholders and researchers collaboratively mapped infrastructural, cultural and literacy-related barriers to existing AI health consultation tools in resource-limited settings. **b**, Cocreation: Prototype development of the GPT-4-powered P&P Care (OpenAI; GPT-4o mini) chatbot prioritized low-literacy interfaces, integrated with e-learning modules to address AI health literacy deficits. **c**, Testing and refinement: The codesign team iteratively tested and refined the prototype models via codesigned evaluation metrics and questionnaires, and the virtual patient experiment validated the chatbot performance. The radar plot illustrates the mean scores for health awareness and communication quality metrics (attention, listenability, conciseness, integrity and empathy) between e-learning plus and consultation-only groups from the virtual patient experiments, with error bars representing the s.d. **d**, Implementation and evolution: Two on-site pilot studies (urban/rural) refined real-time speech-to-text integration and enhanced chatbot functionality in suboptimal network connectivity. The codesign process shaped the pragmatic RCT’s design, including context-aligned trial arms, patient-centred evaluation rubrics and culturally appropriate recruitment. Base map from Ministry of Civil Affairs of the People’s Republic of China (map approval no. GS (2022)1873). The original base map data are unaltered; colours have been modified for illustrative purposes. This map is schematic only.

To bridge this gap in literacy and access, we developed P&P Care (Population Medicine and Public Health), an LLM-powered primary care chatbot for community settings, using a dual-track role-play codesign framework (Fig. 1). This codesign framework enabled: (1) community-aligned chatbot development ensuring usability across diverse populations; (2) synergistic integration of multimedia e-learning modules to enhance interaction quality; and (3) the design and implementation of a pragmatic randomized controlled trial (RCT) to evaluate the role of e-learning in boosting health awareness and consultation efficacy (Methods). We demonstrate how the codesign framework can effectively deploy an LLM-driven solution to address literacy barriers and digital accessibility, advancing patient self-care and health awareness in resource-limited settings.

## Results

### Community codesign

Engaging community codesign members from 11 Chinese provinces through four iterative phases—contextual understanding, cocreation, testing and refinement, and implementation and evolution (Table 1)—directly shaped the P&P Care chatbot architecture, e-learning modules and pragmatic trial design.

**Table 1:** Dual-track role-play codesign

Codesign	Tracks	Descriptions	Role-play activities and objectives
Contextual understanding	Community	Identify and prioritize unmet needs, sociocultural barriers and infrastructural constraints in existing digital health tools.	Assume researcher roles to codify key challenges and delineate culturally relevant success metrics.
	Researcher	Map technical capabilities/constraints of LLMs and establish ethical/regulatory compliance requirements.	Adopt user personas to simulate real-world interactions with existing chatbots, documenting usability gaps.
Cocreation	Community	Codevelop interaction modules with researchers, leveraging community expertise.	Lead prototype ideation workshops, prioritizing accessibility for low-literacy populations.
	Researcher	Translate community-derived requirements into technical specifications.	Conduct ethical impact assessments to mitigate risks.
Testing and refinement	Community	Provide qualitative feedback on interaction experiences with developed prototypes.	Codefine patient-centred evaluation metrics.
	Researcher	Iteratively refine prototypes, using community-informed simulation testing and performance audits to optimize functionality and usability.	Perform heuristic evaluations to proactively identify and address technical and experiential limitations.
Implementation and evolution	Community	Execute community-led pilot deployments to identify context-specific implementation barriers.	Analyse usage patterns to iteratively refine feedback instruments and optimize implementation protocols.
	Researcher	Conduct real-time equity audits of LLM outputs to address demographic performance gaps.	Stress-test system resilience via simulated high-load scenarios in resource-constrained environments.

#### P&P Care chatbot

The Initial contextual understanding phase (Beijing, September–October 2024) involved 20 patients and caregivers, as well as 16 community health workers (Extended Data Table 1). Through storytelling, focus groups and on-site demonstrations, participants identified key barriers to effective AI interaction, including difficulties in initiating dialogues, correcting chatbot errors, managing consultation duration and directing the conversation flow (Extended Data Table 2). These insights informed a dual-pronged strategy: (1) developing an LLM-powered chatbot to minimize demands on patient AI health literacy and (2) creating supplementary e-learning modules to address needs beyond the chatbot’s immediate capabilities (Fig. 1a).

During cocreation, community stakeholders shaped patient-centric symptom articulation protocols, while researchers engineered these inputs into an adaptive dialogue algorithm optimized for low-literacy contexts (Fig. 1b). These optimizations reduced AI health literacy demands by 66.7% (12/18, pre–post analysis), with all pilot users (8/8 users) reporting enhanced comprehension during simulated consultations.

In the testing and refinement phase, iterative refinements using community-defined rubrics (Supplementary Table 1) achieved target benchmarks (≥3.5/5) across all metrics except health awareness (Fig. 1c), confirming the necessity of complementary e-learning modules. Usability testing confirmed an 81.3% (26/32, among community participants who previously reported anxiety in interaction) reduction in preconsultation anxiety and 97.2% (35/36) adherence to interaction protocols. All 1,200 simulated patient interactions demonstrated 100% safety compliance and 100% hallucination-free interactions.

In the implementation and evolution phase, a pilot study (20 patients, Beijing) revealed a 60% (12/20) preference for voice interfaces among older/low-literacy users, driving integration of real-time speech-to-text conversion (Fig. 1d). A subsequent pilot study in rural areas (50 patients, the remaining 10 provinces) exposed infrastructural constraints, with consultation completion rates plummeting to 40% (4/10) in mountainous regions (Sichuan/Chongqing). Four engineering optimizations—tiered service backbones, offline caching and offline functionality, adaptive latency handling and network bandwidth optimization—resolved these bottlenecks, achieving 98.43% completion in ablation testing (Supplementary Table 2).

#### E-learning modules

**Extended Data Fig. 1:**
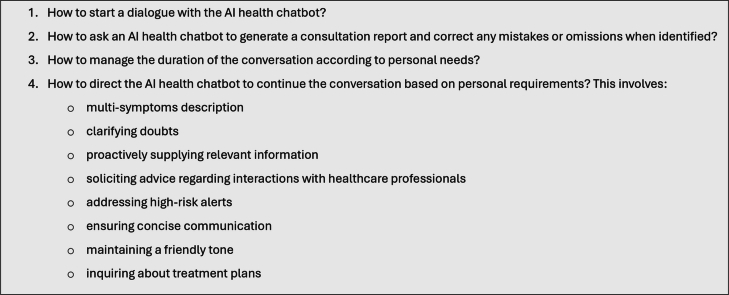
**Key barriers identified in the contextual understanding phase**.

The e-learning modules were developed following the initial chatbot testing to address the co-identified interaction barriers (Extended Data Fig. 1). Our interdisciplinary team codesigned the educational framework, solicited feedback from the 36 community stakeholders and used a voting mechanism to prioritize community preferences and improve content and accessibility.

Participatory cocreation workshops revealed that older adults required explicit guidance for initiating interactions, leading to a short video tutorial on dialogue initiation (Fig. 1b). Patients’ role-play activities prioritized preferences for bite-sized videos, leading to the development of concise (≤30 s) multimedia video tutorials. Quantitative mapping showed that 94.4% (34/36) of community stakeholders preferred modular navigation over fixed sequences, which informed a nonlinear content structure. Through three iterative prototyping cycles, stakeholders defined four core interaction modules: interaction initiation, session closure, time management and personalized content autonomy—each optimized to reduce cognitive load while preserving user agency (Supplementary Table 3).

In a virtual patient experiment (Fig. 1c), the e-learning modules significantly improved users’ objective health awareness (mean difference 1.25 ± 0.09, *P* < 0.001; Extended Data Table 2) and consultation efficiency (attention, mean difference 1.03 ± 0.11, *P* < 0.001).

#### Trial arms and endpoint selection

Logistical constraints precluded a conventional, usual care control arm owing to extreme primary care physician shortages and provider reluctance to record routine practices over privacy and workload concerns. Consequently, the RCT compared P&P Care with integrated e-learning versus without, supplemented by a comparative analysis of de-identified usual care and telemedicine dialogues after the trial. The primary outcome, objective awareness of health needs, was selected to measure patients’ capacity for active engagement in managing their health via the chatbot.

### Participant flow and baseline data

**Fig. 2:**
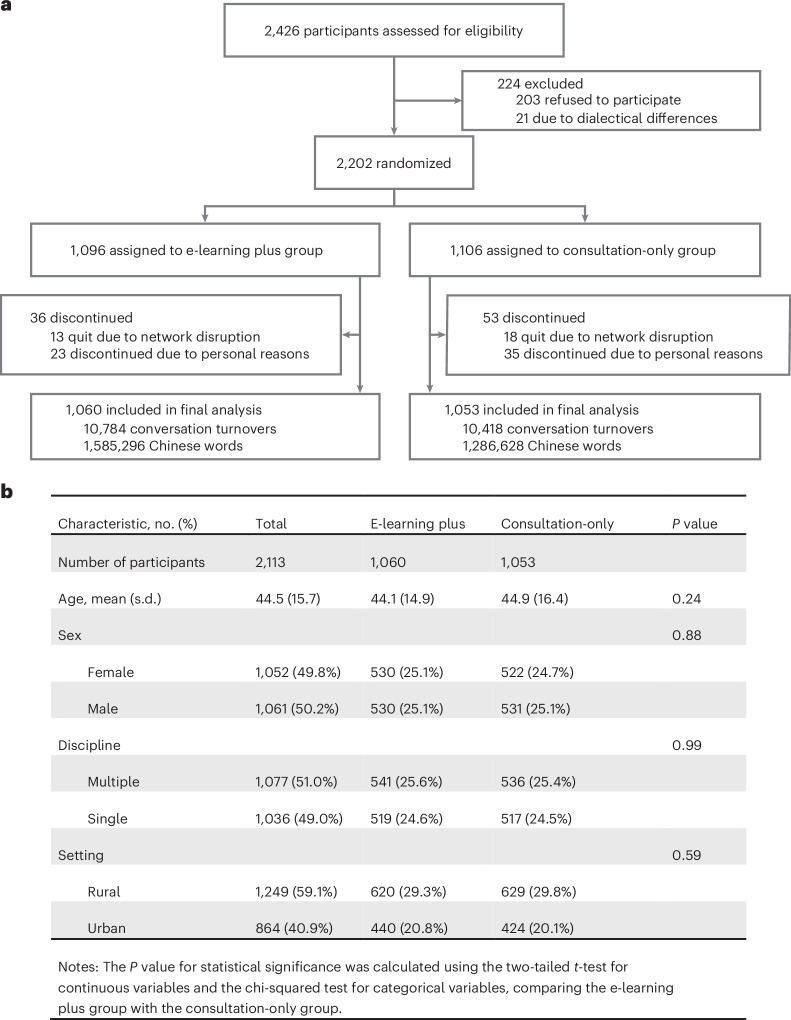
Participant flow and baseline demographics in the RCT. **a**, Flow diagram depicting the participant recruitment, randomization and follow-up procedure. **b**, Panel summarizing the participants’ demographics, demonstrating covariate balance across the e-learning plus and consultation-only groups.

A total of 2,426 community residents were evaluated for eligibility, with 224 participants either opting out or being excluded for various reasons (Fig. 2a). This left 2,202 participants who were randomly assigned to the e-learning plus group (*n* = 1,096) or the consultation-only group (*n* = 1,106) for 1:1 allocation. Among these, 58 participants later chose to opt out for personal reasons, and 31 quit due to network disruptions (Fig. 2a). Proportions of participant dropouts due to network instability were comparable between rural and urban communities (urban 1.03% versus rural 1.66%; *P* = 0.30) and between western and eastern China (eastern 1.34% versus western 1.48%; *P* = 0.91). Cultural and linguistic adaptation in the codesigned recruitment process proved critical: codesigned recruitment scripts reduced voice interface attrition to 9.3% (21/224).

Our trial analysis included 2,113 participants, with 1,060 from the e-learning plus group and 1,053 from the consultation-only group. The population had a mean age of 44.5 (standard deviation (s.d.) 15.7), with a nearly equal sex distribution (1,052 females, 49.8%; 1061 males, 50.2%). A majority (1,249; 59.1%) resided in rural areas, with the remainder (864, 40.9%) in urban settings. Over half of the participants (1,077; 51.0%) required consultation across more than one ICD-10 (International Classification of Diseases, 10th Revision) domain. Detailed demographics are shown in Fig. 2b, and age distribution is presented in Supplementary Fig. 1.

As prespecified, a corpus of 21,202 de-identified conversational turns (comprising 2,871,924 Chinese words) was collected for blinded evaluation. This included 10,784 turns (1,585,296 words) from the e-learning plus group and 10,418 turns (1,286,628 words) from the consultation-only group. Participants in the e-learning plus group generated significantly longer conversations (1,495.6 ± 1,134 words) than those in the consultation-only group (1,221.9 ± 706.3 words; *P* < 0.001). After adjusting for age, sex, community setting (rural/urban) and ICD-10 domain consulted (single/multiple), e-learning exposure remained significantly associated with both conversational turns (coefficients 0.28; *P* = 0.02) and total word count (coefficients 270.76; *P* < 0.001). All P&P care responses underwent both automated and human evaluation, demonstrating 100% safety compliance and 100% hallucination-free outputs.

### Awareness of health needs

**Fig. 3:**
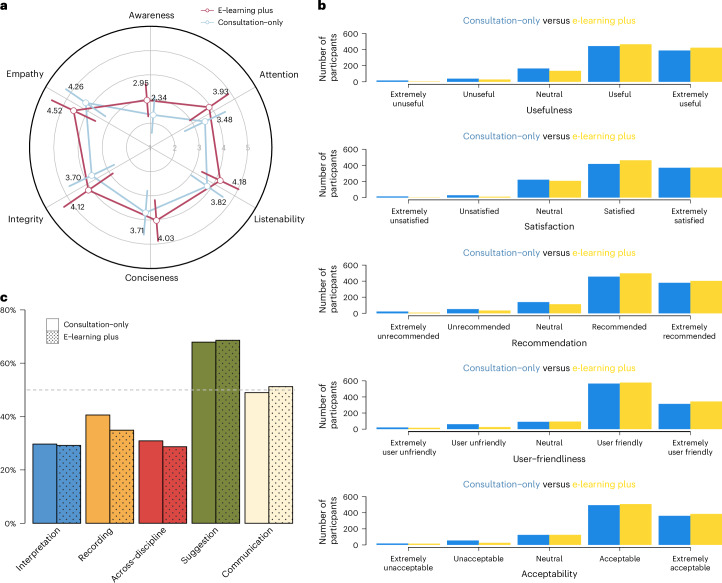
E-learning plus versus consultation-only on health awareness, communication quality and perceived value from the RCT. **a**, A radar plot illustrating the mean scores for health awareness and communication quality metrics (attention, listenability, conciseness, integrity and empathy) with error bars representing the s.d., demonstrating the significant differences in health awareness and communication quality between the e-learning plus (*n* = 1,060 participants) and consultation-only (*n* = 1,053 participants) groups. **b**, The distribution of patient feedback across five dimensions (usefulness, satisfaction, user-friendliness, recommendation and acceptability), showing the perceived value and user experience of two groups. **c**, Participants reported the most valued features of P&P Care chatbot among those who believed them more useful than their current primary care. These features include: interpretation (diagnostic report interpretation), recording (efficient medical history elicitation and documentation), across ICD-10 domains (simultaneous consultation of multiple ICD-10 domains), suggestion (preliminary diagnostic suggestions) and communication (enhanced patient–physician communication skills).

The primary outcome was the objective awareness of health needs assessed through patient dialogues with P&P Care and was significantly higher in the e-learning plus group (2.95 ± 1.22) than in the consultation-only group (2.34 ± 1.02; *P* < 0.001; Fig. 3a). The e-learning plus group also showed superior performance across the secondary outcomes on consultation quality: attention (3.93 ± 0.99 versus 3.48 ± 0.99; *P* < 0.001), defined as the capacity to align consultation duration with participant expectations; listenability (4.18 ± 0.84 versus 3.82 ± 0.91; *P* < 0.001), reflecting accurate percetion of participant needs; conciseness (4.03 ± 0.84 versus 3.71 ± 0.79; *P* < 0.001), assessing the clarity of exchanges; integrity (4.12 ± 0.96 versus 3.70 ± 0.92; *P* < 0.001), evaluating information completeness and comprehensiveness; and empathy (4.52 ± 0.67 versus 4.26 ± 0.75; *P* < 0.001), measuring the friendliness and respectfulness to participants. Raw effect sizes of the e-learning intervention across all six evaluation dimensions were substantial (Extended Data Table 2).

Participants in both the e-learning plus (4.21 ± 0.79) and consultation-only (4.09 ± 0.90) groups reported high perceived usefulness of P&P Care for understanding their health conditions compared with their usual primary care (Fig. 3b). The e-learning plus group showed statistically significant enhancements compared with the consultation-only group across multiple dimensions: perceived usefulness (4.21 ± 0.79 versus 4.09 ± 0.90; *P* = 0.006; Extended Data Table 3), user-friendliness (4.14 ± 0.80 versus 4.04 ± 0.89; *P* = 0.009), consultation satisfaction (4.13 ± 0.77 versus 4.05 ± 0.89; *P* = 0.030), perceived effectiveness of the consultation report in facilitating physician communication (recommendation: 4.18 ± 0.82 versus 4.06 ± 0.94; *P* = 0.006) and likelihood of future utilization (acceptability: 4.15 ± 0.83 versus 4.07 ± 0.91; *P* = 0.033).

Among participants who perceived P&P Care consultations as more beneficial than their current primary care services, the feature of providing initial diagnoses was the most appreciated (e-learning plus: 68.6%, 611/891; consultation-only: 67.9%, 566/833; Fig. 3c), followed closely by enhancing physician–patient communication (e-learning plus: 51.2%, 456/891; consultation-only: 49.0%, 408/833).

### Demographic differences

**Fig. 4:**
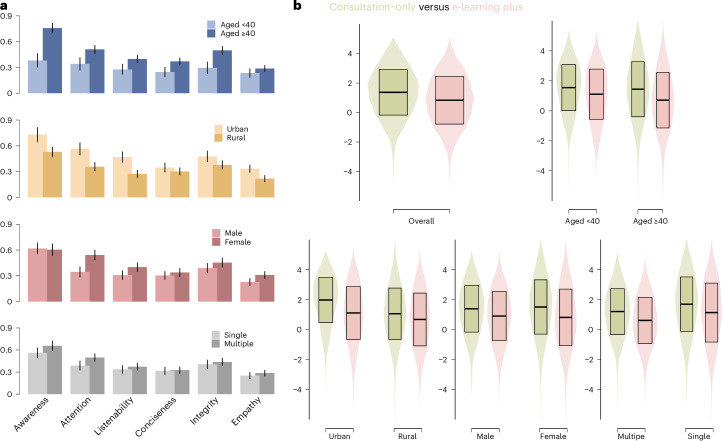
Subgroup analysis of e-learning plus versus consultation-only on health awareness, communication quality and health awareness gap. **a**, Differential effects on health awareness and communication quality. The bar plot shows the raw effect size across demographic subgroups. Error bars represent the s.d. (*n* = 2113 participants, sample sizes for each subgroup are provided in Fig. 2b). Between-group comparison statistics were estimated using group-level data presented in Extended Data Table 3 and independent sample *t*-tests. **b**, Health awareness gap. The violin plot shows distributions of the health awareness gap (defined as the difference between self-reported and objective health awareness) across subgroups. Boxes within violins represent the mean (centre line) and the mean ± s.d. (box bounds; *n* = 2,113 participants). Sample sizes for each subgroup are provided in Fig. 2b.

Stratified analyses revealed that e-learning conferred disproportionately greater benefits to specific participant subgroups. Older participants demonstrated a substantially higher increase in health awareness following e-learning compared with younger participants (aged ≥40 years, 0.76 ± 0.06 versus aged <40 years, 0.38 ± 0.08; Fig. 4a). While baseline awareness was comparable between older and younger participants in the consultation-only group, e-learning substantially augmented awareness in older individuals (Extended Data Table 4). Furthermore, older participants exhibited greater improvements in listenability, attention, conciseness, integrity and empathy following e-learning. These findings suggest that e-learning effectively enhances health awareness and communication quality among older adults.

Rural participants exhibited smaller improvements across all six evaluation metrics compared with their urban counterparts (Fig. 4a), with the most notable disparities in awareness (rural 0.53 ± 0.06 versus urban 0.73 ± 0.08), attention (0.36 ± 0.05 versus 0.57 ± 0.07) and listenability (0.27 ± 0.05 versus 0.47 ± 0.06). These findings indicate that e-learning yielded diminished gains in rural settings, potentially reflecting variations in engagement with e-learning. The participants’ feedback aligned with this contrast: rural participants were less likely to place an extremely high value on their consultation experiences than urban participants (Supplementary Fig. 2).

Female participants achieved comparable gains in health awareness to male participants but showed greater gains across the five consultation quality metrics. In addition, participants requiring multidisciplinary care (multiple ICD-10 domains) experienced more substantial benefits than those with single-domain consultations.

While objective health awareness significantly differed between trial arms, self-reported health awareness showed no significant difference (e-learning plus: 3.79 ± 1.06; consultation-only: 3.72 ± 1.13; *P* = 0.12), suggesting a possible misalignment between actual knowledge acquisition and perceived understanding. To quantify the efficacy of e-learning in mitigating this discrepancy, we defined the health awareness gap as the difference between self-reported and objective health awareness. This gap was significantly reduced in the e-learning group compared with the consultation-only group (0.83 ± 1.62 versus 1.37 ± 1.55; *P* < 0.001). The e-learning intervention consistently attenuated this gap across all subgroups, with particularly pronounced effects among older adults, women, urban residents and those with complex care needs, indicating a targeted efficacy in populations with higher knowledge discrepancies (Fig. 4b).

### Quality of dialogue

**Fig. 5:**
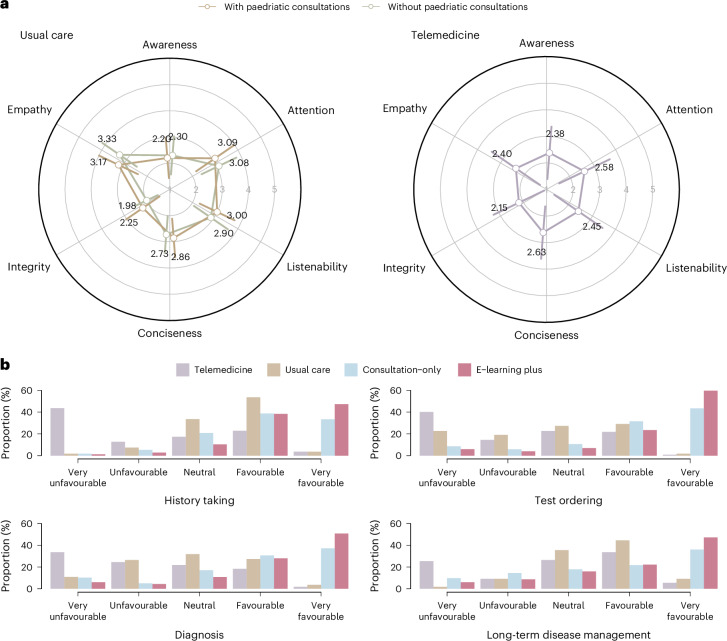
Comparison of P&P Care (e-learning plus versus consultation-only), usual primary care and telemedicine in health awareness, consultation quality and care delivery. **a**, Communication quality of dialogue samples collected from usual care (rural clinics and urban community health centres, *n* = 220 samples) and telemedicine (*n* = 220 samples). The radar plot illustrates the mean scores for health awareness and communication quality metrics (attention, listenability, conciseness, integrity and empathy), with error bars representing the s.d. The usual care plot shows the scores in the presence or absence of paediatric consultation samples. **b**, The distribution of quality scores for history taking, diagnosis, test ordering and long-term disease management across four care models (e-learning plus (*n* = 1,060 samples), consultation-only (*n* = 1,053 samples), usual care (*n* = 110 samples) and telemedicine (*n* = 110 samples)).

To benchmark P&P Care with existing healthcare solutions, we collected 110 dialogue samples from rural clinics and urban community health centres (10 per province; 924 conversation turns and 28,890 Chinese words) with informed consent. The sample included dyadic parent–child communication (18.2%), a demographic that was underrepresented in our RCT. Analysis of these dialogues revealed considerably lower communicative quality scores compared with dialogues with P&P Care, regardless of inclusion or exclusion of paediatric consultations (Fig. 5a). Qualitative assessment identified two key distinctions: first, conventional care frequently incorporated informal, non-clinical discourse (6.5% of dialogues), reflecting established patient–provider relationships absent in P&P Care interactions; second, clinicians routinely performed physical assessments (39.1% of cases, for example, auscultation and blood pressure measurement) based on patient reported symptoms—a capability beyond P&P Care’s language-dependent interface.

Similarly, we collected 110 primary care dialogue samples from the HaoDF telemedicine platform (10 per province; 373 turns of conversation and 17,152 Chinese words). Evaluation of these dialogues again showed lower communicative quality scores compared with P&P Care dialogues (Fig. 5a).

### Quality of primary care

We further compared P&P Care with usual care and telemedicine across four domains central to primary care quality: history-taking, diagnostic precision, test ordering appropriateness and long-term disease management. P&P Care (e-learning plus variant) significantly outperformed both usual care and telemedicine across all domains (*P* < 0.001; Fig. 5b), with marked improvements in diagnosis (4.13 ± 1.15 versus usual care: 2.86 ± 1.05; telemedicine: 2.30 ± 1.17) and test ordering (4.27 ± 1.13 versus usual care: 2.68 ± 1.17; telemedicine: 2.29 ± 1.23; Extended Data Table 5). P&P Care also surpassed consultation-only chatbot interactions (*P* < 0.001), underscoring the value of integrated e-learning modules.

## Discussion

We developed P&P Care, a patient-facing LLM-powered primary care chatbot, using a dual-track role-play codesign framework designed to enable equitable LLM deployment. In a RCT across 11 provinces in China, this community-codesigned chatbot, which integrates literacy-bridging e-learning alongside technical and cultural adaptations, provided high-quality primary care consultations. The codesign approach effectively engaged underserved populations by-passed by conventional telemedicine and hospital-centric LLM applications, proving essential for harnessing LLMs in resource-limited settings. While tested in China, this approach may offer a globally scalable model to augment primary care capacity, addressing the critical gap between the rapid AI advances and their equitable real-world deployment^25^.

Our RCT established that the integrated e-learning intervention significantly enhanced participants’ objective health awareness compared with consultation alone. This gain was accompanied by marked improvements in consultation quality, indicating that structured preconsultation education effectively primes patients for richer and more productive AI interactions. These results align with evidence that building patients’ knowledge and confidence for engagement could amplify the utility of digital health tools^26,27^. Substantial improvements across demographical groups further underscore the synergistic potential of combining educational and interactive LLM components in resource-limited communities. Ultimately, by pairing preparatory education with interactive LLM consultations, P&P Care moves beyond a static decision-support tool to function as a dynamic interface for people-centred care.

Both trial arms reported high perceived usefulness of P&P Care relative to standard primary care. However, the e-learning plus group demonstrated superior scores in perceived usefulness, user-friendliness, satisfaction and future utilization intent, suggesting that even incremental improvements in consultation quality could greatly enhance patient engagement^28^. Furthermore, while P&P Care provided immediate responses, telemedicine consultations exhibited substantial delays, with human doctors often responding hours or days after patient inquiries. Participants particularly valued features that supported initial diagnoses and improved subsequent physician communication, highlighting P&P Care’s role in facilitating timely interventions and effective care coordination^29^.

Subgroup analyses reveal a need for tailored implementations, with older adults (≥40 years) deriving considerably greater benefits from the e-learning intervention. This group exhibited substantial improvements in objective health awareness and consultation quality, in line with evidence that older populations who often face steeper health literacy gradients could derive disproportionate advantage from structured educational support for digital tools^30,31^. These results challenge the stereotype of low digital adaptability in older adults^28^, instead positioning them as a high-impact demographic for this e-learning integrated version.

Conversely, the reduced impact of e-learning in rural populations underscores persistent systemic barriers to digital engagement^32^. While e-learning narrowed geographic disparities by converging post-intervention health awareness, its suboptimal uptake in rural areas suggests a need for more context-specific adaptations, such as hybrid digital–in-person education models. Sex-based analyses revealed a nuanced pattern: while health awareness gains were comparable between genders, women demonstrated greater improvements in consultation quality, potentially reflecting gendered patterns in communication and engagement with educational content^33^. Furthermore, participants requiring multidisciplinary consultations derived greater benefits, suggesting that e-learning could effectively facilitate the navigation of complex care pathways—a critical function in fragmented systems grappling with rising multimorbidity^8,9^.

A key finding was the discrepancy between objective and self-assessed health awareness, a recognized challenge in digital health where perceived and actual knowledge are misaligned^34^. The e-learning intervention significantly reduced this health awareness gap, particularly among older individuals, women, urban residents and those seeking multiple ICD-10 domain consultations, suggesting its role in recalibrating self-assessment accuracy. This is clinically crucial, as overconfidence may delay care-seeking, whereas underconfidence may induce unnecessary anxiety^35,36^. By narrowing this gap, the integrated e-learning version may foster judicious health decisions; further studies should determine its effects on real-world care utilization.

Although randomization balanced baseline characteristics, the internal validity of our trial may be subject to several limitations. The extended preparation time for the e-learning plus group might introduce performance bias, potentially affecting self-reported satisfaction. However, this risk was mitigated by blinded assessment of objective endpoints. Low and balanced attrition across groups minimized potential attrition bias. We were unable to monitor participant adherence to the e-learning modules; full compliance might yield greater gains in health awareness. The reduced benefit of e-learning among rural participants compared with urban participants highlights the importance of addressing factors that affect e-learning uptake in rural communities. Finally, the chatbot audio system incorporates over 200 Chinese dialects; its limited representation of certain regional variants may disproportionately affect rural and ethnic minority populations in autonomous regions who rely on audio interfaces.

While our trial demonstrates the efficacy of the community-codesigned chatbot in improving primary care consultation, scaling this approach requires overcoming several hurdles. Beyond infrastructural limitations and linguistic heterogeneity, public hesitancy, particularly among marginalized populations, highlights the need for culturally sensitive, equity-focused adaptation^37^. Furthermore, regulatory uncertainties on accountability and data privacy present adoption hurdles, demanding agile governance frameworks for LLM applications^38^. Finally, strategic alignment of AI infrastructural investments with local healthcare priorities is essential to avoid exacerbating existing health inequities^39^.

Our use of a collection of primary care recordings as a proxy for usual care, a necessary choice due to clinician reservations about real-time recording, probably introduced self-selection bias. This proxy may represent a higher-than-average quality of care, creating a conservative comparator that probably underestimates the benefits of P&P Care. Despite this, P&P Care demonstrated significantly superior communicative quality compared with this benchmark. Future RCTs directly comparing P&P Care with real-time usual care are needed to precisely quantify implementation effectiveness. Finally, while this study focused on initial deployment challenges and short-term outcomes in resource-limited settings, rigorous longitudinal studies are essential to evaluate the long-term health outcomes of chatbot-assisted consultations versus usual care.

Despite these limitations, the community-codesigned P&P Care demonstrates robust functionality in resource-limited environments, confirming its practical implementation potential across rural China. The platform maintained low and comparable rates of conversation disruption across diverse urban and rural network settings. Our analysis indicates that P&P Care outperforms both standard in-person visits and telemedicine in core primary care consultations. Enhancements in diagnostic precision and test ordering suggest a potential to reduce delays and diagnostic errors for high-burden conditions in underserved areas. Furthermore, its capabilities in comprehensive history-taking and chronic disease management address systemic care fragmentation, while its user-friendly design more effectively engages vulnerable populations with digital literacy barriers than conventional telemedicine. Operationally, the chatbot’s autonomous handling of community patients’ consultations presents a viable strategy to mitigate primary care workforce shortages.

The success of this codesign approach in rural China, where physician shortages reduce care to transactional medication dispensing^40^, suggests the framework’s potential to address global structural gaps in resource-limited settings. The dual-track process revealed that communities prioritized care continuity (for example, longitudinal health guidance over episodic consultations), a need that conventional LLM systems fail to meet. By engaging community stakeholders and researchers in reciprocal role-playing to develop context-aware solutions, the approach helped to align the chatbot with the lived realities of the communities it serves. As global health systems confront rising demands, this codesign framework offers a viable path to operationalize the World Health Organization (WHO)’s vision of people-centred care, illustrating how LLMs can augment the human dimensions of healing.

## Methods

### Study design

We developed P&P Care, an LLM-powered primary care chatbot using a dual-track role-play codesign framework. The framework delineates roles and responsibilities for community stakeholders (patients, caregivers and community health workers, including village doctors) and research stakeholders (public health experts, primary care physicians and LLM engineers) to ensure parity in influence. A core innovation is reciprocal role-play, wherein stakeholders simulate one another’s perspectives; for example, researchers simulated patient interactions under low-literacy constraints while community members critiqued prototypes from a designer’s viewpoint. This approach mitigates common limitations of traditional codesign, such as tokenism and power imbalances.

We evaluated the codesigned system of the P&P Care chatbot and its integrated literacy-bridging e-learning modules in a parallel RCT. The trial was conducted across 11 geographically and culturally diverse Chinese provinces, encompassing both rural and urban communities. Study sites were selected to represent China’s tiered healthcare development, spanning eastern (Beijing, Shandong), central (Hubei, Shanxi) and western (Chongqing, Gansu, Shaanxi, Sichuan, Guangxi, Inner Mongolia and Xinjiang; Fig. 1d) regions. For reporting participant dropouts due to network instability, Hubei was reclassified with the eastern group because of its comparable infrastructure, while Shanxi was grouped with the western provinces. The inclusion of three of China’s five autonomous regions (Guangxi, Inner Mongolia and Xinjiang) ensured representation of major ethnic minority populations with distinct linguistic and cultural backgrounds. The trial included community residents who were randomly assigned to receive a consultation with preparatory e-learning (e-learning plus) or without (consultation-only) in a 1:1 ratio. The primary outcome was the objective awareness of health needs. All primary care consultations with the chatbot were recorded, along with participant feedback to assess consultation quality. Trial reporting adheres to the CONSORT-AI guidelines^41^.

### Ethical approval

The study protocol received approval from the Ethics Review Committee of the Chinese Academy of Medical Sciences and Peking Union Medical College and was prospectively registered with the Chinese Clinical Trial Registry (identifier ChiCTR2500098101). All participants provided informed consent in accordance with the Declaration of Helsinki, with explicit disclosure that this is an exploratory experiment and that the chatbot health advice given during consultations should not be used for disease management without clinician oversight. Stringent data protection protocols were implemented in this study, ensuring that all data were anonymized and encrypted for privacy protection.

### Codesign process

#### Contextual understanding

To understand community needs and existing technological limitations, we initiated codesign workshops involving 36 community stakeholders (12 patients, 8 caregivers and 16 health workers from Beijing) and 8 role-playing researchers (from Peking Union Medical College; Extended Data Table 1). Three community health worker liaisons (with more than 6 years of community engagement experience) and four researchers, each with expertise in specific areas (digital healthcare, public health, primary care and LLM techniques), facilitated the codesign process. This phase involved the iterative interaction with and evaluation of three prevalent AI health consultation platforms in China: XiaoHeJianKang (TikTok), XunFeiXiaoYi (Tencent) and LingYiZhiHui (Baidu), through periodic in-person meetings, virtual conferences (WeChat and Tencent Meeting) and collaborative online documentation (WPS 365).

In the community track, stakeholders articulated unmet needs and contextual challenges in utilizing existing health consultation chatbots (for example, difficulties with technical terminology and verbose responses) by methods including storytelling, focus groups and on-site demonstrations. They engaged in role-playing as researchers to define key challenges and desired improvements for a user-friendly interface (for example, a WeChat mini-program). In the research track, researchers defined LLM capabilities and limitations, and established compliance requirements and ethical guidelines (for example, aligning with WHO principles for ethical AI in primary care). Researchers role-played as users to interact with existing chatbots, directly experiencing limitations such as health literacy barriers and jargon-heavy outputs.

Qualitative data generated from this process underwent reflexive thematic analysis to pinpoint critical limitations in current AI tools. The initial codebook was created from preliminary responses and iteratively refined through ongoing analysis cycles. Two independent researchers coded transcripts, achieving high interrater reliability (Fleiss’ kappa *κ* = 0.80); all discrepancies were resolved through consensus. Community participants, particularly older adults, expressed a critical need for explicit guidance on initiating dialogues with chatbots, articulating concerns regarding uncertainty at the interaction onset and the potential for providing inaccurate information, as exemplified by the direct quote: “I am at a loss know what to do at the start, I do not know what the chatbot is asking for, and I worry that I fail to provide the correct information to ruin the consultation.” In addition, a majority (8/12) of patients valued longitudinal disease management above episodic symptom resolution, with one stating: “I want to know how I could live with the disease in my remaining life, not just address symptoms today.”

#### Cocreation

The cocreation phase produced P&P Care prototypes and e-learning modules through parallel, role-playing tracks. Community stakeholders (patients, caregivers and health workers) contributed their lived experiences to design interaction modules. Through role-play, they acted as designers to ensure usability and cultural appropriateness, shaping features such as bite-sized video content and culturally relevant health recommendations. Researchers (physicians, public health experts and LLM engineers) translated community needs into technical solutions. Adopting user personas, they role-played low-literacy interactions to evaluate social feasibility, focusing on language simplification and culturally sensitive delivery.

The chatbot was developed using systematic prompt engineering through an iterative two-step process: role-specific fine-tuning and ethical-operational refinement, ensuring alignment with Chinese primary care guidelines and WHO ethical AI principles.

For role-specific fine-tuning, we implemented a bidirectional dialogue architecture with two clinical reasoning stages. Bidirectional interactions referred to proactive interaction, where the chatbot not only responded to users’ questions but also proactively inquired about them. During the inquiry stage, the chatbot was trained through Reinforcement Learning from Human Feedback^42^ to engage patients in active, multiturn dialogues to elicit comprehensive health-related information aligned with primary care standards, including demographics, medical history, presenting symptoms, living environment, lifestyle behaviours and psychological status. In the conclusion stage, the chatbot was trained to generate one to three differential relevant diagnosis possibilities, each accompanied by supporting or refuting evidence (for example, symptom alignment and risk factors) and suggest preventative care actions (for example, screenings and lifestyle changes). Furthermore, the chatbot was trained to suggest one to three actionable recommendations for physical/laboratory examinations, with rationales tied to clinical guidelines (for example, HbA1c testing for prediabetes screening in individuals with a high body mass index), through Chain of Thought prompting^43^. Real-time pop-up alerts from a health risk detector ensured user awareness of potential health risks. In addition, chatbot response generation incorporated a robust verification pipeline: all outputs sequentially cleared a safety filter (Llama2-Guard, confidence >0.5)^44^ and a hallucination detector (SelfCheckGPT, mean contradiction probability <0.5)^45^. Failed checks triggered regeneration (up to three attempts) before a fallback.

For the ethical and operational refinement, we used prompt augmentation and agent techniques to refine the model^46^. A multidisciplinary feedback-and-refinement team (36 community stakeholders, 2 primary care physicians, 2 public health experts and 1 AI-ethics-trained graduate student) iteratively interacted with the model via adversarial testing, prioritizing (1) safety and hallucination—avoiding harmful, biased or non-compliant outputs (for example, rejecting inappropriate treatment suggestions); (2) patient-centredness—enhancing empathetic communication (for example, probing unmet needs); and (3) context adaptability—optimizing performance for low-resource settings (for example, simplifying language for low-literacy users).

To effectively address users’ needs following their e-learning experience, we instructed an LLM-driven agent to assist the counsellor chatbot in providing appropriate responses. This agent is designed to understand the intent behind patient inquiries through intention analysis, specifically following e-learning modules 2.a, 4.f and g in Supplementary Table 3. The prompts were tailored as responses to the specific e-learning modules, and the outputs from the agent were structured in JSON format for straightforward parsing and further processing by the chatbot.

#### Testing and refinement

During the testing and refinement phase, community stakeholders engaged directly with prototypes through role-play, adopting researcher perspectives to define evaluation metrics on consultation quality (Supplementary Table 1). Rubrics of attention (matching consultation duration with participant expectations) and conciseness (clarity and brevity in conversations) surfaced from community participants role-playing as researchers during the codesign workshops, in response to the question, “If you could design a chatbot for health conversation, what aspects would you value most?”.

Meanwhile, researchers conducted simulated patient interactions to identify technical limitations, such as integrating visual aids for literacy adaptation. Model refinement integrated two complementary approaches: human interaction via our codesign team to ensure contextual relevance, and simulated patient interactions leveraging the Chinese MedDialog dataset containing 3.4 million dialogues across 172 ICD-10 domains. Virtual patient behaviours were informed by community-derived interaction patterns through a process we term community-informed virtual patient interaction.

For chatbot training, we developed a community-informed patient agent that emulated realistic patient behaviours using synthetically generated profiles structured via a knowledge graph architecture^47^. The graph formalized patient attributes as interconnected nodes with schema definitions encoded via the Resource Description Framework^48^, populated using Simple Protocol and Resource Description Framework Query Language queries^49^. We synthesized 1,200 unique virtual patient profiles (see Supplementary Fig. 2 for illustrations), with 50% requiring interdisciplinary consultation, all validated by five board-certified clinicians through consensus validation. The patient agent emulated behavioural patterns derived from community interactions, including dialogue hesitancy and ambiguous symptom articulation, with termination protocols ending interactions when virtual patients acknowledged query resolution.

A randomly selected subset of 600 profiles refined the chatbot’s prompt templates using a codesigned rubric evaluated by a blinded panel of five experts and two community laypersons. A score below 4 triggered refinement.

For e-learning module refinement, the remaining 600 patient profiles were allocated to e-learning plus or consultation-only groups. In the e-learning plus group, the patient agent internalized educational modules through a JSON-structured decision model, while the consultation-only group initiated dialogues with deactivated education. Initial dialogues from 300 profiles were evaluated by the expert panel to identify suboptimal elements, prompting iterative content revisions prioritizing clarity and WHO primary care communication guideline alignment. The remaining 300 profiles, unexposed to prior refinement, served for validation and sample estimation, assessed by the same panel using the codesigned rubric with anonymized, random case assignment.

#### Implementation and evolution

During the implementation and evolution phase, our dual-track approach facilitated continuous refinement through role-playing exercises and two localized pilot studies (20 patients, Beijing, November–December 2024; and 50 patients, the remaining 10 provinces, January–February 2025). Community stakeholders identified critical implementation barriers—including network limitations, impracticality of usual care controls and culturally specific recruitment needs—while analysing usage patterns to develop network attrition mitigation strategies and refine feedback questionnaires for capturing nuanced communication outcomes. Concurrently, researchers conducted computational audits to detect demographic performance disparities, expanding dialectal training corpora based on these findings. Through role-playing as users in resource-constrained scenarios, researchers stress-tested system resilience, validating the chatbot’s operational capacity in low-infrastructure environments.

In pilot studies, primary outcome and secondary outcomes related to consultation quality were the same as those from the testing and refinement phase. Secondary outcomes on patient consultation experiences were structured on the basis of patients’ qualitative feedback via thematic analysis. Qualitative data on the consultation experience, derived from eight iterative role-play workshops, underwent reflexive thematic analysis to establish patient-centred evaluation metrics. The initial codebook was developed from preliminary responses and iteratively refined through successive analysis cycles. Two independent researchers coded transcripts, achieving high interrater reliability (Fleiss’ kappa *κ* = 0.86); all discrepancies were resolved through consensus. The emergent themes, perceived usefulness, interface user-friendliness, satisfaction, future use intention and facilitation of physician communication, were subsequently operationalized into a 5-point Likert scale survey. This questionnaire underwent three codesign iterations with community stakeholders to enhance its relevance and clarity. The questionnaires’ internal consistency was robust (Cronbach’s *α* > 0.70 for all dimensions), and face validity was established through iterative feedback from 20 community residents, ensuring relevance to community primary care contexts. The finalized survey instrument is presented in Supplementary Table 4.

Four engineering optimizations addressed network constraints: (1) implementing a tiered service backbone that dynamically adjusts context window size and output length on the basis of network latency; (2) using local caching to enable offline functionality; (3) designing adaptive latency handling through extended timeouts and decoupled processing; and (4) prioritizing text transmission to optimize bandwidth. We evaluated performance under severe network throttling simulating worst-case rural conditions: internet protocol instability (10% probability of switching every 10 s), connection drops (20% probability every 10 s), 60% packet loss, 1,000 ms latency and 100 kilobits per second bandwidth. An ablation study assessing consultation completion rates systematically removed each optimization: tiered service elimination, cached functionality disablement, latency handling deactivation, bandwidth optimization removal and combined strategy ablation.

### Trial design

#### Participants

Participants in the trial must demonstrate a need for health consultation or express a willingness to engage in LLM-based health consultations. Moreover, participants were required to be between 20 and 80 years of age. Individuals who presented with psychological disorders or drug abuse and any other conditions that may compromise communicative interactions or the integrity of assessments were excluded.

#### Intervention and comparator

This is a double-blinded, parallel group trial. Participants were randomly assigned to either the e-learning plus or the consultation-only group upon enrolment. All participants were informed that they would go through two phases: preparation for the consultation and then participation in health consultations. In the e-learning plus group, participants received training on AI health literacy through the e-learning modules during the preparation phase before engaging in health consultations. In the consultation-only group, participants underwent controlled preparation, where the e-learning modules were deactivated, before engaging in health consultations. During the preparation phase, participants were required to recall the symptoms and conditions of their recent and current illnesses. They then conducted health consultations with the P&P Care chatbot (see Supplementary Tables 5 and 6 for illustrations). After consultation, participants completed a post-consultation questionnaire to provide feedback on their experience.

#### Outcomes

The primary outcome measures objective awareness of health needs. Secondary outcomes included attention, integrity, listenability, conciseness and empathy (Supplementary Table 1). These outcomes serve as objective indicators of the consultation dialogue and were evaluated by the same panel involved in the chatbot development phase, who were blinded to group assignment. All assessments were conducted post-recruitment, with one trained assessor assigned to each dialogue. The secondary outcomes also include self-reported awareness of health needs, satisfaction, usefulness, user-friendliness, acceptability and recommendation, which were retrieved from feedback questionnaires provided by the participants (Supplementary Table 4). Additional outcomes include the number of conversational turns and the average number of words per participant case.

#### Sample size

Sample sizes were estimated based on the difference in health awareness between the e-learning plus and consultation-only groups. Using data from preliminary virtual experiments, we calculated that a sample size of 1,200 participants would be required to achieve 80% power at a significance level of 0.05. To account for inflated literacy scores in virtual patients compared with real-world community residents, we conducted an interim analysis after enrolling 500 participants. This adjustment necessitated recalculating the differences, requiring a revised sample size of 2,000 participants to maintain statistical power.

#### Recruitment

Our local codesigned teams, who lived in the area and were proficient in the local dialect, assisted with participant recruitment and the trial. The codesigned team proactively contacted potential community participants who needed LLM-based health consultations or were willing to try these consultations. For those who indicated interest, the teams provided comprehensive descriptions of the study, emphasizing that it is exploratory and that any advice rendered by P&P Care serves solely as a reference and should not be utilized as a definitive basis for disease therapy. Participants received an informed consent form before enrolment and had the opportunity to ask questions. After this process, potential participants who met the established inclusion and exclusion criteria were formally recruited. The community engagement track facilitated iterative collaboration with stakeholders across localized pilot studies, identifying the need for culturally appropriate recruitment strategies. Recruitment took place from 4 to 30 March 2025.

#### Randomization and blinding

We used individual-level parallel randomization without stratification, utilizing a computer-generated random sequence for participant assignment to each experimental group. We implemented allocation concealment to maintain the confidentiality of the random allocation and minimize bias. Throughout the intervention and analysis phases, the group information and operational materials remained undisclosed to all participants and researchers.

#### Statistical methods

To ensure objectivity, data collection and subsequent statistical analyses were conducted by independent researchers. We assessed the normality of scale value distributions across 11 dimensions and used two-sample *t*-tests with unequal variances for intergroup comparisons where appropriate. For dimensions that exhibited significant skewness, we used non-parametric Mann–Whitney *U* tests. All statistical tests were two-tailed, with a significance threshold set at *P* < 0.05. In addition, we applied the Benjamini–Hochberg adjustment for multiple testing corrections based on the total number of tests performed. R 4.3.0 was used to perform the statistical analyses and present the results.

Subgroup analyses were performed on the basis of age, community setting, sex and ICD-10 domains. To match ICD-10 domains with the dialogue data, we collaborated with local primary care workers to create a comprehensive list of 23 ICD-10 domains (Supplementary Table 7). We then prompted a language model to match each dialogue with the identified ICD-10 domains. One dialogue could be assigned to more than one ICD-10 domain. The accuracy of the assignment was corroborated by a panel consisting of five medical professionals.

To establish a contextual benchmark for evaluating the P&P Care chatbot, we prospectively collected 110 dialogue samples (10 per province) from existing primary care encounters in townships and communities following informed consent protocols. In addition, we collected 110 primary care dialogue samples (10 per province) from the telemedicine platform (HaoDF). Because these contextual dialogues frequently incorporated informal exchanges characteristic of established patient–physician relationships, which differed substantively from the interactions facilitated by P&P Care and were readily discernible, they were maintained as a separate dataset and not integrated with P&P Care patient dialogues for blinded evaluation. Each dialogue underwent independent evaluation by two trained members of the same evaluation panel. Interrater reliability was rigorously assessed and confirmed using Fleiss’ kappa statistics (*κ* > 0.80).

Furthermore, we expanded our analysis to compare P&P Care with usual care and telemedicine across four domains central to primary care quality: (1) history-taking, (2) diagnostic precision, (3) test-ordering appropriateness and (4) long-term disease management. Blinded evaluations by independent expert clinicians (five board-certified primary care physicians) assessed conversation dialogues using validated 5-point Likert scales for completeness, appropriateness and clinical relevance, according to standardized evaluation guidelines (Supplementary Table 8). Interrater reliability for these assessments was high (*κ* > 0.80).

### Reporting summary

Further information on research design is available in the Nature Portfolio Reporting Summary linked to this article.

## Supplementary information

Supplementary Information Supplementary Tables 1–8 and Figs. 1–3; Study protocol and statistical analysis plan (English); Study protocol and statistical analysis plan (Chinese).

Reporting Summary

## Figures and Tables

**Extended Data Table 1:**
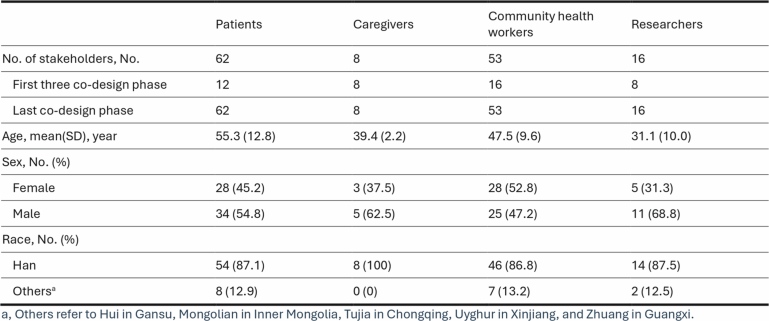
Demographic breakdown of stakeholders in co-design process

**Extended Data Table 2:**
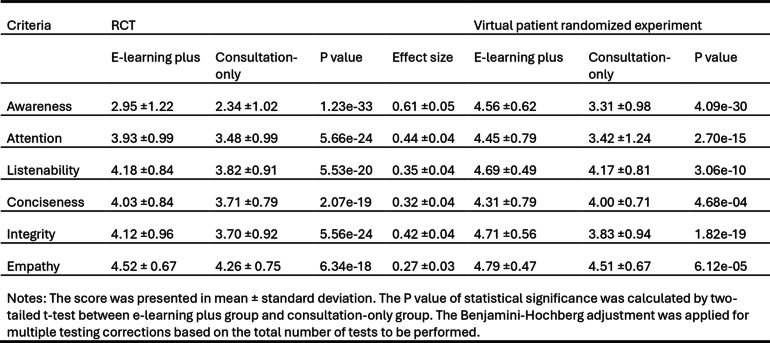
Health awareness and consultation quality in the RCT and the virtual patient randomized experiment

**Extended Data Table 3:**
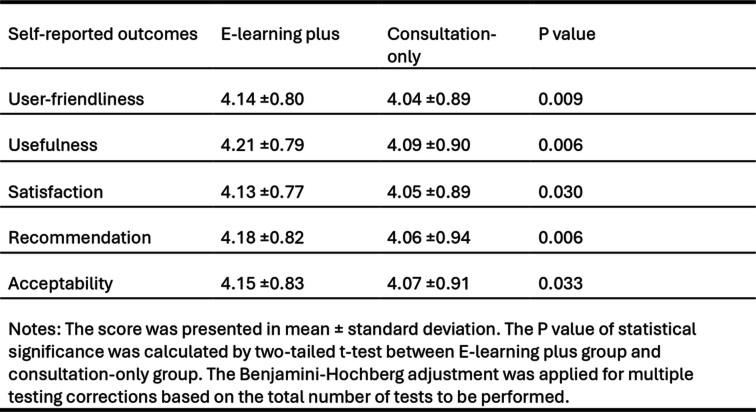
Particpant reported secondary outcomes in the RCT

**Extended Data Table 4:**
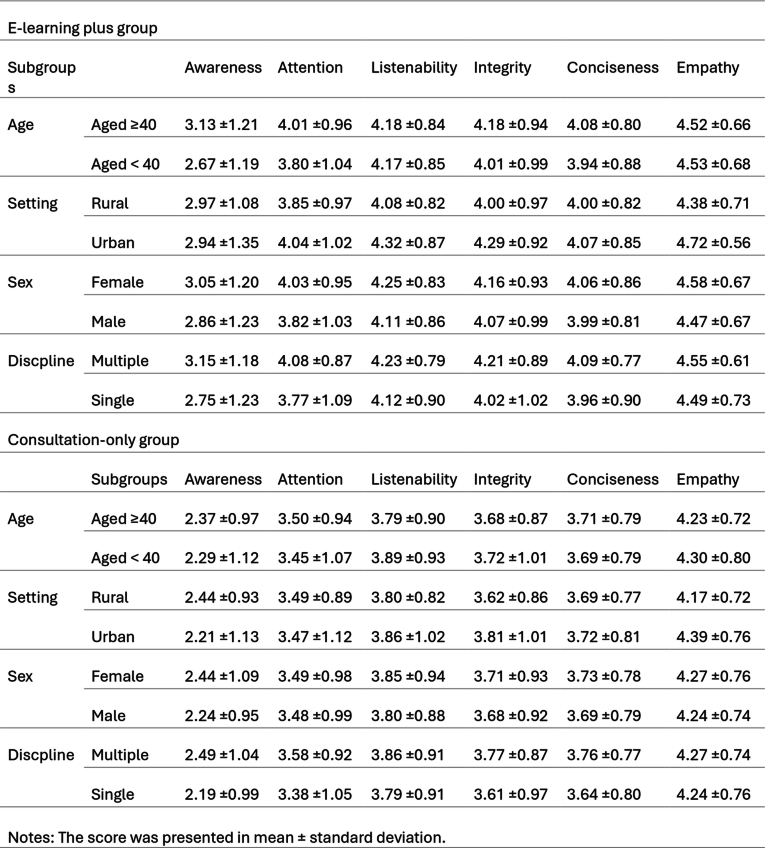
Demographic differences in the RCT

**Extended Data Table 5:**
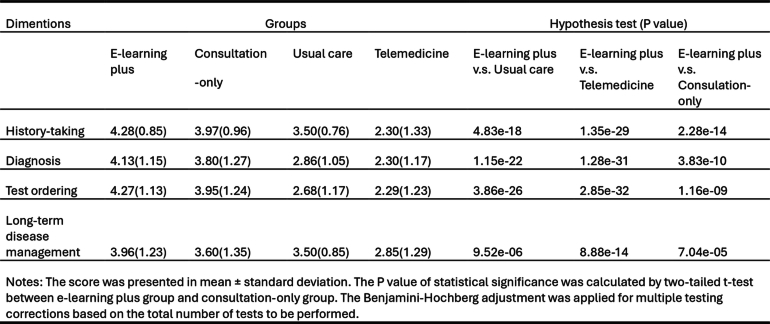
Quality of primary care

## Data Availability

The study protocol is provided in the Supplementary Information. Source data are provided in Extended Data Tables 2–5 and are available via GitHub at https://github.com/ShashaHan-collab/PaPCare-CommunityRCT (ref. ^50^). Raw conversation data are not publicly available due to the need to protect participant privacy, in accordance with the ethical approval for this study. Anonymized, non-dialogue individual-level data underlying the results can be requested by qualified researchers for academic use. Requests should include a research proposal, statistical analysis plan and justification for data use and can be submitted via email to S.H. (hanshasha@pumc.edu.cn). All requests will be reviewed by the Chinese Academy of Medical Sciences and Peking Union Medical College. Applicants will receive an initial response within 2 months, and approved requests will be granted access via a secure platform after execution of a data access agreement.

## Code availability

Comparative statistical analyses are detailed in the Article. Code for data visualization is available via GitHub at https://github.com/ShashaHan-collab/PaPCare-CommunityRCT (ref. ^50^).
