# Supplementary material for: A community-codesigned LLM-powered chatbot for primary care: a randomized controlled trial
Source: Nat Health. 2026 Jan 19;1(2):238–50. doi: 10.1038/s44360-025-00021-w (PMC12880911; doi:10.1038/s44360-025-00021-w)
Supplement: Supplementary file 1 — Supplementary Tables 1–8 and Figs. 1–3; Study protocol and statistical analysis plan (English); Study protocol and statistical analysis plan (Chinese). [file 44360_2025_21_MOESM1_ESM.pdf]

nature health

Supplementary information https://doi.org/10.1038/s44360-025-00021-w

A community-codesigned LLM-powered chatbot for primary care: a randomized controlled trial

In the format provided by the authors and unedited

Supplementary Tables and Figures

Table of Contents

Supplementary Table 1 The scoring criteria 2

Supplementary Table 2 Results of the throttling test in the ablation studies 5

Supplementary Table 3 Schematic of e-learning modules 6

Supplementary Table 4 The survey questionnaires 8

Supplementary Table 5 A de-identified case of P&P care chatbot-patient consultation in the RCT: Acute episodes 11

Supplementary Table 6 A de-identified case of P&P care chatbot-patient consultation in the RCT: Multimorbidity conditions 15

Supplementary Table 7 The list of ICD 10 domains 19

Supplementary Table 8 Conversation evaluation rubrics on primary care delivery 20

Supplementary Fig. 1 Age distribution of participants in the RCT. 22

Supplementary Fig. 2 Distribution of patient feedback across five domains by rural and urban residents. 23

Supplementary Fig. 3 An illustrative example of a virtual patient profile. 24

Supplementary Table 1 The scoring criteria

**Citeria** (Establisehd in which co-design phases)	**Description**	**Performance levels**
Awarenss (Testing & Refinement)	During the conversation, how clear was the patient about their own health needs?	Very unclear, the patient simply answered the questions given
		Unclear, the patient mostly answered the questions given and chatted a little
		Okay, in addition to answering the questions, the patient occasionally asked about their health proactively
		Quite clear, the paitent often asked about their health proactively
		Very clear, the patient always asked about their health proactively and pointed out any problems with the responses
Attention (Testing & Refinement)	How did the consultation time met the patients' demanding time?	Extremely unmet
		Unmet
		Okay
		Well met
		Highly met
Listenability (Testing & Refinement)	Was the AI counselor able to listen and accurately identify the patients' needs?	Completely misunderstood, the AI counselor 's responses were completely irrelevant to the user's needs
		Rarely understood, the AI counselor 's responses didn't match the user's needs very well

		Somewhat understood, the AI counselor 's responses often deviated from the user's needs
		Most of the time understood, although occasionally the recognition was not accurate or comprehensive
		Completely understood, the AI counselor said exactly what the user wanted to know
Conciseness (Testing & Refinement)	Was the inquiry and response of the conversation concise and clear?	Very wordy, the long-winded explanations were tiring for the patient to read
		Quite wordy, especially during long conversations, it was a bit tiring for the patient to read
		Okay, mostly concise but sometimes wordy, making it difficult for the patient to find the main points
		Relatively concise, occasionally a bit wordy but still acceptable
		Very concise and clear, even when the content was long, it was well-organized and the patient could quickly grasp the main points, making it easy to read
Integrity (Testing & Refinement)	Was the information given by AI counselor comprehensive and complete?	Not comprehensive at all, there were serious omissions and oversights
		Mostly incomplete, there were often omissions and oversights
		Okay, but there were still some omissions and oversights
		Mostly comprehensive, occasionally there were omissions but they could be corrected after being pointed out
		Very comprehensive, and it also provided additional useful information
Empathy		Not friendly at all, the chatbot only asked questions mechanically and ignored the patient's feedback

(Testing & Refinement)	Was the AI counselor friendly and respectful towards the patient?	Often unfriendly, the AI counselor wouldn't explain the user's doubts
		Okay, the AI counselor would take the initiative to explain complex medical terms to the user
		Most of the time it was friendly, and the AI counselor could explain most of the user's doubts
		Very friendly, the AI counselor could patiently explain all the user's doubts

Notes: The initial Chinese questionnaires have been translated into English and carefully reviewed to maintain their original meaning. Specifically, the principal investigator (SH) translated the Mandarin text into English. Three researchers (SL, SZ, and XT) compared the English version and the Chinese version line by line to resolve semantic and cultural discrepancies.

Supplementary Table 2 Results of the throttling test in the ablation studies

Network environment sensitive design	Proportions of successful consultation completion
P&P AI Care engineering optimization	98.43%
Removing the tiered service backbone	90.25%
Eliminating local caching and offline functionality	65.50%
Disabling adaptive latency handling	70.17%
Deactivating network bandwidth optimization	76.63%
Ablating all the four adaption engineering strategies	43.60%

Notes: The engineering optimization framework of the P&P Care platform exhibited robust performance, achieving a 98.43% success rate in simulated adverse network conditions. Ablation studies revealed that the tiered backbone architecture was crucial for maintaining resilience against IP switching and connection drops, with its removal resulting in an 8.18% performance reduction. The absence of local caching and offline functionality led to a dramatic decrease in success rate to 65.50%, highlighting its critical role in mitigating packet loss and intermittent connectivity by minimizing real-time data dependencies. Adaptive latency handling, through dynamic response buffering aligned with user tolerance thresholds, contributed a 28.26% improvement. Furthermore, bandwidth optimization, employing traffic prioritization and data compression in low-throughput environments, accounted for a 21.8% gain. These findings demonstrate the system's stability and availability under extreme network stress, underscoring the importance of these architectural considerations for ensuring equitable access to AI-driven healthcare in resource-constrained settings.

Supplementary Table 3 Schematic of e-learning modules

1. Dialogue initiation control. Please start the conversation in the following ways:

   Describe your specific health condition: I have a problem with my [body part]. For example, “I have a stomachache.”

2. Dialogue termination control. Please end the conversation by using the following method to obtain a summary consultation report and review it:
   a. **Request a summary report:** Please generate a summary consultation report based on our conversation.
   b. **Correct errors in the report:** If you find any discrepancies or missing information in the report, please point out the specific issues and ask AI to regenerate the report.
3. Dialogue duration control:
   a. **To end the conversation:** Directly use the method in Step 2. to end the conversation.
   b. **To continue the conversation:** Ask specific questions about the content you want to learn more about.
4. Dialogue content control:
   a. **Gradual symptom description**: If you have discomfort in multiple body parts, describe each symptom step-by-step to avoid omissions. Avoid inputting all symptoms at once.
   b. **Clarifying doubts**: If you have any questions about the AI's questions or answers, feel free to ask for clarification. For example, if asked about vestibular rhinitis and you're unsure what it is, you can ask, "What is vestibular rhinitis?"
   c. **Proactively supply relevant information**: If there are any aspects of your health that haven't been asked about, feel free to provide them proactively, such as medical history, lifestyle, and living environment.
   d. **Advice regarding interactions with physicians**: If you need medical advice, you can proactively ask for it. For example, you can ask, "What advice would you give me for communicating with a doctor?"
   e. **Addressing high-risk alerts**: If a high-risk alert is triggered, make your own judgment based on your situation. If you feel there is no risk and want to continue the conversation, you can ask specific questions about the content you want to learn more about. If you're uncertain about the risk, you can ask for more information regarding the high-risk alerts.

f.	**Concise expression**: If you feel the AI's questions or answers are too wordy, you can ask it to express itself more concisely. For example, you can say, "Please be concise."
g.	**Friendly tone**: If you feel the AI's questions or answers are unfriendly, you can ask it to express itself more politely. For example, you can say, "Please be more friendly."
h.	**Preliminary treatment plans**: If you want to understand a preliminary diagnosis and treatment plan, you can continue to ask for relevant advice. This can include suggestions for tests and examinations, medication, rehabilitation therapy, exercise prescriptions, daily precautions, and lifestyle recommendations. For example, you can ask, "What precautions should I take daily?"

Notes: The initial Chinese texts have been translated into English and carefully reviewed to maintain their original meaning.

There are a total of thirteen instructions, each of which is accompanied by two options as outlined below:

- Of course! I am well acquainted with this and will ensure to follow up during the consultation.
- I am uncertain about how to proceed; please provide assistance.

The thirteen instructions tailored to address their individual needs were provided to participants as a personalized learning experience. After reviewing each instruction, participants were prompted to provide their responses. If they selected the first option, they would be guided to the subsequent instruction. Conversely, if they chose the second option, they would receive a video demonstration that explicitly illustrated how to implement the instructions on our consultation platform. This instructional process persisted until all thirteen instructions had been comprehensively learned.

Supplementary Table 4 The survey questionnaires

Citeria (Establisehd in which co-design phases)	Description	Performance levels
Self-awarenss (Implementation & Refinement)	During your conversation, how clear were you about your own needs?	Very unclear, I simply answered the questions it gave me
		Unclear, I mostly answered the questions it gave me and chatted a little
		Okay, in addition to answering its questions, I occasionally asked about my health proactively
		Quite clear, I often asked it about my health proactively
		Very clear, I always asked it about my health proactively and pointed out any problems with its responses
Satisfaction (Testing & Refinement)	Overall, how satisfied were you with the consultation service?	Very dissatisfied
		Dissatisfied
		Okay
		Quite satisfied
		Very satisfied
User-friendliness (Testing & Refinement)	How easy was it to have a conversation with the P&P Care counselor?	Very difficult, I had no idea how to start a conversation
		Difficult, it often asked questions that were hard to understand, and I didn't know how to respond

		Neutral
		Pretty easy, it was easy to talk to it
		Very easy, it was more relaxed and easy to talk to it than to a doctor
Usefulness (Implementation & Refinement)	Compared to primary care services in townships and communities, how helpful was the consultation service in helping you initially understand your condition?	Not helpful at all, the difference is too big
		Not very helpful, the difference is big
		Hard to say, the questioning angle is different from primary care doctors in townships and communities
		Quite helpful, it's about the same
		Very helpful, it considers more comprehensively than primary care doctors in townships and communities
	[If the answer is "Quite helpful" or "Very helpful", please select all that apply:] What did you find most helpful or impressive about this consultation? (Multiple choice)	Interpreting diagnostic reports
		Efficiently inquiring and recording medical history
		Allowing consulting multiple medical domains simultaneously
		Providing preliminary diagnosis suggestions
		Helping me learn how to communicate better with doctors
Acceptability (Testing & Refinement)	If you get sick or worried about your health next time, would you be willing to use P&P Care	Definitely not, there’s no need
		Not really, I’m not very familiar with using it yet

	to get a preliminary understanding of your health problem?	Uncertain, it’s hard to say
		Yes, so I can communicate better with the doctor
		Definitely, so I can communicate better with the doctor and even avoid unnecessary visits to the hospital
Recommendation (Implementation & Refinement)	If doctors could use the report given by P&P Care to understand your condition in advance, do you think it would make communication between doctors and patients easier?	Definitely not, doctors don’t need to know in advance and prepare
		Not really, it’s hard for doctors to get a preliminary understanding of my condition this way
		Uncertain, it’s hard to say
		Yes, it would help doctors get a preliminary understanding of my condition
		Definitely, it would help doctors understand my condition and ask more comprehensive and targeted questions

Notes: The initial Chinese questionnaires have been translated into English and carefully reviewed to maintain their original meaning. Specifically, the principal investigator (SH) translated the Mandarin text into English. Three researchers (SL, SZ, and XT) compared the English version and the Chinese version line by line to resolve semantic and cultural discrepancies.

Supplementary Table 5 A de-identified case of P&P care chatbot-patient consultation in the RCT: Acute episodes

P&P Care: I'm P&P Care. What can I do for you?
Patient: My right shoulder's been hurting.
P&P Care: Could I ask if you're male or female? And roughly how old are you?
Patient: Male, aged 48.
P&P Care: Thanks for that. Can you tell me what the pain feels like? Like, is it sharp, dull, or something else? Does anything make it better or worse?
Patient: It hurts when I press on it, and it feels kinda weak.
P&P Care: Got it. How long's this been going on? Any particular activity that might've caused it, like exercise or heavy lifting?
Patient: About a week now. Started after I moved some heavy boxes.
P&P Care: Thanks. Besides the shoulder pain and weakness, any other issues? Headaches? Trouble moving? Anything else bothering you?
Patient: The pain's messing with my sleep.
P&P Care: I understand, poor sleep really takes a toll. What's your living situation like? Any dampness or big temperature changes where you live?
Patient: Not really, no.
P&P Care: Thanks. How about your daily routine? Does your work keep you in one position a lot? Do you exercise regularly?
Patient: I try to walk every day.
P&P Care: Good to know. How's your stress level lately? Any big pressures or mood swings that might be affecting you?
Patient: Nah, nothing major.
P&P Care: Appreciate you sharing. Based on our talk, here are some possibilities:

Shoulder Muscle Strain
Supporting Evidence: You mentioned pain started after lifting heavy objects and hurts when pressed, suggesting possible muscle or soft tissue injury.
Opposing Evidence: No obvious signs of swelling or bruising.
Frozen Shoulder (Adhesive Capsulitis)
Supporting Evidence: Weakness and pain could be related to inflammation around the shoulder joint, especially if worsened by activity.
Opposing Evidence: Short symptom duration; frozen shoulder typically has a longer history.
Cervical Spine Issues (e.g., Cervical Disc Herniation)
Supporting Evidence: Cervical problems can sometimes cause shoulder pain and weakness.
Opposing Evidence: You haven't mentioned neck symptoms or numbness.
Next, I recommend the following examinations for a more accurate assessment:
Shoulder X-ray: To check for signs of fracture or arthritis.
MRI or Ultrasound: To help evaluate soft tissue, muscle, and joint injuries.
Blood Tests: To assess inflammation markers and understand if there's an underlying inflammatory response.

Please consider these suggestions and let me know your thoughts.

Patient: Actually, my neck's been feeling kinda sore too.
P&P Care: Since you mentioned neck soreness, we should be extra cautious. Just so we're clear on potential risks: This could relate to cervical issues like disc problems or muscle strain. Does the soreness get worse when you move? Any numbness or tingling?
Patient: A little bit, yeah.
P&P Care: Thanks. That numbness/tingling could point more toward a cervical issue. Let's consider:

Cervical Muscle Strain
Supporting Evidence: Soreness and pain after lifting heavy objects suggest possible muscle fatigue from overuse.
Opposing Evidence: The presence of numbness may not fully align with a diagnosis of muscle strain alone.
Cervical Disc Herniation
Supporting Evidence: Neck soreness and accompanying numbness/tingling could be related to compression of a cervical disc.
Opposing Evidence: No clear neurological symptoms or severe pain reported.
Cervical Spondylosis
Supporting Evidence: Long-term neck discomfort and occasional numbness fit the presentation of cervical spondylosis.
Opposing Evidence: Need further information on whether other complex symptoms exist (e.g., arm weakness).
To better understand your condition, I recommend the following examinations:
Cervical Spine X-ray: To assess cervical spine structure and check for issues like bone spurs.
Cervical Spine MRI: Can clearly view cervical discs and soft tissues, helping confirm if there's nerve compression.
Nerve Conduction Velocity Test: To evaluate nerve function, especially given your numbness.

Please consider these suggestions. What do you think? Also, are there specific situations in your daily life where the pain worsens, or patterns to its relief?

Patient: You think my neck and shoulder pain are connected?
P&P Care: Very possible. Neck and shoulder structures are closely linked. Here are some possible links:

Nerve Compression: Cervical spondylosis or disc herniation can compress nerve roots, causing shoulder pain and arm discomfort.
Muscle Tension: Neck muscles influence shoulder muscles; tension or strain in neck muscles can lead to shoulder pain and discomfort.
Referred Pain: Sometimes, pain originating in the neck can radiate to the shoulder, making you feel pain there even though the source is the neck.

Considering your symptoms, I recommend detailed examinations to clarify the issues in both the cervical spine and shoulder. Would you like me to suggest further treatment options, or do you have additional symptoms or questions?

Patient: Any exercises that might help?
P&P Care: Try these gentle movements to ease discomfort and improve flexibility:

Neck Exercises:
Neck Side Bend: Sit upright, slowly bring your right ear towards your right shoulder, hold for 15-30 seconds, then switch sides. Repeat 2-3 times per side.
Neck Flexion/Extension: Sit upright, slowly bring your chin towards your chest (flexion), hold 15-30 seconds. Then slowly lift your head, looking upwards (extension), hold 15-30 seconds. Repeat 2-3 times.
Shoulder Exercises:
Shoulder Circles: Stand or sit upright, slowly rotate shoulders forwards, upwards, backwards, and downwards in a circular motion. Do 10 circles in each direction.
Shoulder Stretch: Extend one arm straight across your body. Use your other hand to gently pull the elbow towards your body. Hold for 15-30 seconds. Switch sides. Repeat 2-3 times per side.
Relaxation Exercises:
Deep Breathing: Sit down, relax shoulders. Inhale deeply, feeling your chest and abdomen expand, then exhale slowly. Repeat 5-10 times.
Small Range Movements: Get up and move around periodically. Do simple stretches to avoid staying in one position for too long.
Precautions:
Start slowly, avoiding excessive stretching or force.
Stop immediately if any exercise increases your pain, and consult a doctor.
Consider physical therapy under professional guidance.

What do you think of these suggestions? Is there a specific exercise or activity you'd like to know more about?

Patient: Can I get a summary?
P&P Care: Health Assessment Summary Report

Patient Information:
Gender: Male
Age: 48 years old
Chief Complaint: Right shoulder pain, neck soreness, accompanied by weakness and sleep disturbance.

Symptom Description:
Nature of Pain: Shoulder hurts when pressed, with weakness; neck soreness with mild numbness and tingling.
Symptom Duration: Approximately 1 week, triggered by lifting heavy objects.
Impact on Life: Pain affects sleep quality.

Possible Diagnoses:
Cervical Muscle Strain: Due to muscle fatigue from lifting heavy objects.
Cervical Disc Herniation: Neck soreness with numbness suggests possible disc involvement.
Cervical Spondylosis: Long-term neck discomfort and occasional numbness.

Recommended Examinations:
Cervical X-ray: To assess cervical structure and potential bone/joint issues.
Cervical MRI: To detail cervical discs and soft tissues, evaluating possible nerve compression.
Nerve Conduction Velocity Test: To assess nerve function and determine if nerves are compressed.

Exercise Recommendations:
Neck side bends, flexion/extension: Enhance neck flexibility.
Shoulder circles, shoulder stretches: Relieve shoulder muscle tension.
Deep breathing: Aid relaxation.
Small range movements: Avoid prolonged static postures.

Conclusion:
Your neck and shoulder pain may be caused by multiple factors, including muscle fatigue from lifting heavy objects and potential underlying cervical spine issues. It is recommended to undergo the suggested examinations for a clear diagnosis and to begin appropriate exercises to alleviate symptoms. Seek prompt medical attention if symptoms worsen or new symptoms appear.

If you have other questions or need further assistance, please let me know.

Patient: That's all. Thank you.

Notes: The initial Chinese questionnaires have been translated into English and carefully reviewed to maintain their original meaning. Specifically, the principal investigator (SH) translated the Mandarin text into English. Three researchers (SL, SZ, and XT) compared the English version and the Chinese version line by line to resolve semantic and cultural discrepancies.

Supplementary Table 6 A de-identified case of P&P care chatbot-patient consultation in the RCT: Multimorbidity conditions

P&P Care: I'm P&P Care. What can I do for you?
Patient: My legs feel uncomfortable when walking.
P&P Care: Let's figure this out. First, are you male or female?
Patient: Male, 78 years old.
P&P Care: Thanks. You mentioned your legs feel uncomfortable when walking. Could you describe what this discomfort feels like? For example, is it pain, soreness, swelling, or something else?
Patient: I just walk really slowly now. Hard to lift my legs properly.
P&P Care: When did this start? Any trigger, like a fall, injury, or health issue?
Patient: Been years. Had knee surgery before, too.
P&P Care: Got it. Tell me about the surgery - when and why? Did you do rehab afterward?
Patient: Both knees replaced a few years back. Still can't walk right.
P&P Care: Understood. You've had both knees replaced, but still experience walking discomfort. Do you have any other health issues, such as hypertension, diabetes, or heart disease? Also, what is your current lifestyle like regarding diet, exercise, and psychological state?
Patient: Yeah, I have hypertension, and had a stroke years ago, too.
P&P Care: Important context. A history of hypertension and stroke can impact your overall health. Are you currently taking medication to manage your hypertension? Also, do you have regular check-ups or monitor your blood pressure?
Patient: Yep, take my pills like clockwork.
P&P Care: Good, it's important that your hypertension is controlled with medication. Now, let me ask, in your daily life, do you feel anxious, depressed, or have other psychological concerns?
Patient: No.
P&P Care: Okay, that's important. Based on our conversation, I can list some possible diagnoses:

Post-Knee Surgery Complications
Supporting Evidence: You mentioned bilateral knee replacement surgery and persistent walking discomfort.
Opposing Evidence: If postoperative recovery was good, gait should typically improve.
Peripheral Neuropathy
Supporting Evidence: Leg discomfort and walking difficulty could relate to nerve problems, especially possible post-stroke.
Opposing Evidence: Lower likelihood if no other neurological symptoms (like numbness, tingling) are present.
Circulation Problems
Supporting Evidence: Hypertension could cause poor circulation, affecting gait.
Opposing Evidence: Likelihood decreases if blood pressure is well-controlled.

I recommend the following examinations:
Knee Joint Imaging (e.g., X-ray or MRI): To assess postoperative knee condition and identify other potential issues.
Blood Tests (including glucose, cholesterol, etc.): To evaluate your overall health, especially risks related to hypertension and stroke.
Neurological Function Tests: To rule out neurological issues like peripheral neuropathy.

Please consider these suggestions. Do you feel there's any other information to add?

Patient: Was the previous surgery not done properly?
P&P Care: Post-surgery recovery can be influenced by many factors, including surgical technique, individual differences, and postoperative rehabilitation. While it's possible the surgery didn't achieve the expected outcome, other factors like aging, other health issues, or poor circulation could also be causing the leg discomfort.
To diagnose the specific problem, imaging examinations can help us better understand the condition of your knee joints and whether any postoperative complications exist. If you have concerns, you can discuss the specifics of your surgery and postoperative prognosis with your doctor.
Do you have any other thoughts or questions about this?
Patient: Thank you. My family is thinking of taking me to the hospital for a check-up later. Any suggestions?
P&P Care: I strongly support your family's plan. Going to the hospital for a check-up is an important step in understanding your health. Here are some suggestions for preparation before your hospital visit:

Prepare Medical History Information
Include your past medical history (hypertension, stroke), surgical history, current medications, and any allergies.
Record Symptoms
Before going to the hospital, try to record your discomfort symptoms in detail, such as when they started, severity, and any methods that provide relief.
Ask a Family Member to Accompany You
Having someone accompany you during the visit can help record the doctor's advice and provide support during communication.
Discuss Medical Examination Items
You can discuss the previously mentioned examinations (knee imaging, blood tests, etc.) with the attending doctor.
Maintain a Positive Attitude
Your emotional health is very important for the entire recovery process. Staying optimistic helps in coping better with examinations and subsequent treatment.

Do you have any other questions? Or is there something specific you feel needs attention?

Patient: What should I pay attention to in daily life?
P&P Care: In daily life, some health precautions can help improve your leg condition and maintain overall health. Here are some suggestions:

Healthy Lifestyle Suggestions
Balanced Diet: Eat plenty of fruits, vegetables, whole grains, and healthy fats. Reduce high-salt, high-sugar, and high-fat foods to help control blood pressure.
Moderate Exercise: Perform exercises suitable for you under your doctor's guidance, such as walking, swimming, or water therapy, to promote circulation and strengthen leg muscles.
Regular Blood Pressure Monitoring: Measure your blood pressure regularly and adjust medication as directed by your doctor.
Maintain Good Mental Health: Keep an optimistic and positive attitude towards life. Seek psychological support if necessary.
Avoid Prolonged Sitting: Get up and move around every hour to promote blood circulation and reduce leg discomfort.
Pay Attention to Knee Health: Avoid strenuous activities and heavy lifting to protect your knee joints.
Maintain an Ideal Weight: Keeping your weight within a healthy range can reduce the burden on your joints.

Please adjust these suggestions based on your actual situation to build a good foundation for your health. What are your thoughts on these recommendations, or is there anything else you'd like to know?

Patient: Please generate a summary report for me so I can review it later.
P&P Care: Okay, here is a summary report of your health condition:

Health Assessment Summary Report

Patient Information:
Gender: Male
Age: 78 years old
Main Symptoms: Discomfort when walking, difficulty lifting legs, slow walking speed.

Medical History:
Knee Surgery: Bilateral knee replacement surgery was performed several years ago.
Other Health Conditions:
Hypertension, currently taking prescribed medication.
A history of cerebral infarction (stroke) requires regular monitoring.

Primary Health Concerns:
Possible post-knee surgery complications.
Possible peripheral neuropathy.
Possible circulation problems.

Recommended Examinations:
Knee Joint Imaging (X-ray or MRI): To assess postoperative status and other potential issues.
Blood Tests (glucose, cholesterol, etc.): To assess overall health and risks related to hypertension and stroke.
Neurological Function Tests: To rule out issues like peripheral neuropathy.

Daily Health Recommendations:
Diet: Maintain a balanced diet, reduce intake of high-salt, high-sugar, and high-fat foods.
Exercise: Engage in appropriate exercise as advised by your doctor to strengthen leg muscles and promote circulation.
Blood Pressure Monitoring: Measure blood pressure regularly and follow your doctor's medication adjustments.
Mental Health: Maintain a positive mindset; seek psychological support if needed.
Avoid Prolonged Sitting: Move around every hour to promote circulation.
Weight Management: Maintain an ideal weight to reduce joint burden.

Follow-up Recommendations:
It is recommended to go to the hospital for a detailed examination as soon as possible, as planned by your family.
Discuss the above examination items and lifestyle adjustments with your primary doctor.

Please review this report carefully. If any adjustments or additions are needed, please let me know anytime.

Patient: Thank you.

Notes: The initial Chinese questionnaires have been translated into English and carefully reviewed to maintain their original meaning. Specifically, the principal investigator (SH) translated the Mandarin text into English. Three researchers (SL, SZ, and XT) compared the English version and the Chinese version line by line to resolve semantic and cultural discrepancies

Supplementary Table 7 The list of ICD 10 domains

No.	ICD 10 domains
1	Allergy
2	Cardiovascular
3	Dermatology and Venereology
4	Endocrinology
5	Fever of Unknown Origin Department
6	Gastroenterology
7	Geriatrics
8	Gynecology
9	Nephrology
10	Neurology
11	Neurosurgery
12	Nutrition
13	Oncology
14	Ophthalmology
15	Oral Medicine
16	Orthopedics
17	Otorhinolaryngology
18	Psychiatry
19	Rehabilitation Medicine
20	Respiratory
21	Rheumatology and Immunology
22	Thyroid and Breast Surgery
23	Urology

Supplementary Table 8 Conversation evaluation rubrics on primary care delivery

Citeria	Questions	Single Chocies
History taking	How do you rate the medical history documentation?	Very favorable, includes all critical and relevant information
		Favorable, includes critical historical information but misses some relevant details
		Neutral, includes critical historical information but lacks other important information
		Unfavorable, does not include critical historical information
		Very unfavorable, no content related to history taking
Diagnosis	How would you rate this physician's diagnosis?	Very favorable, includes the most likely diagnosis and all reasonable differential diagnoses
		Favorable, includes the most likely diagnosis and some reasonable differential diagnoses but misses closely related ones
		Neutral, includes the most likely diagnosis but lacks other reasonable differential diagnoses
		Unfavorable, does not include the most likely diagnosis but provides relevant content to help identify it
		Very unfavorable, no content related to the most likely diagnosis
Test ordering	How would you rate the tests and investigations recommended by this physician?	Very favorable, suggested tests are comprehensive and appropriate
		Favorable, suggested tests are not comprehensive but are appropriate
		Neutral, includes the most essential tests but also suggests inappropriate ones

		Unfavorable, does not include the most essential tests
		Very unfavorable, no content related to appropriate tests and investigations
Long-term disease management	How would you rate the long-term diseasemagement adviced recommended by this physician?	Very favorable, advice is comprehensive and appropriate
		Favorable, advice is not comprehensive but are appropriate
		Neutral, includes the most essential health management advice but there are inappropriate ones
		Unfavorable, does not include the most essential health management advice
		Very unfavorable, no content related to appropriate advice

Notes: The initial Chinese questionnaires have been translated into English and carefully reviewed to maintain their original meaning. Specifically, the principal investigator (SH) translated the Mandarin text into English. Three researchers (SL, SZ, and XT) compared the English version and the Chinese version line by line to resolve semantic and cultural discrepancies.

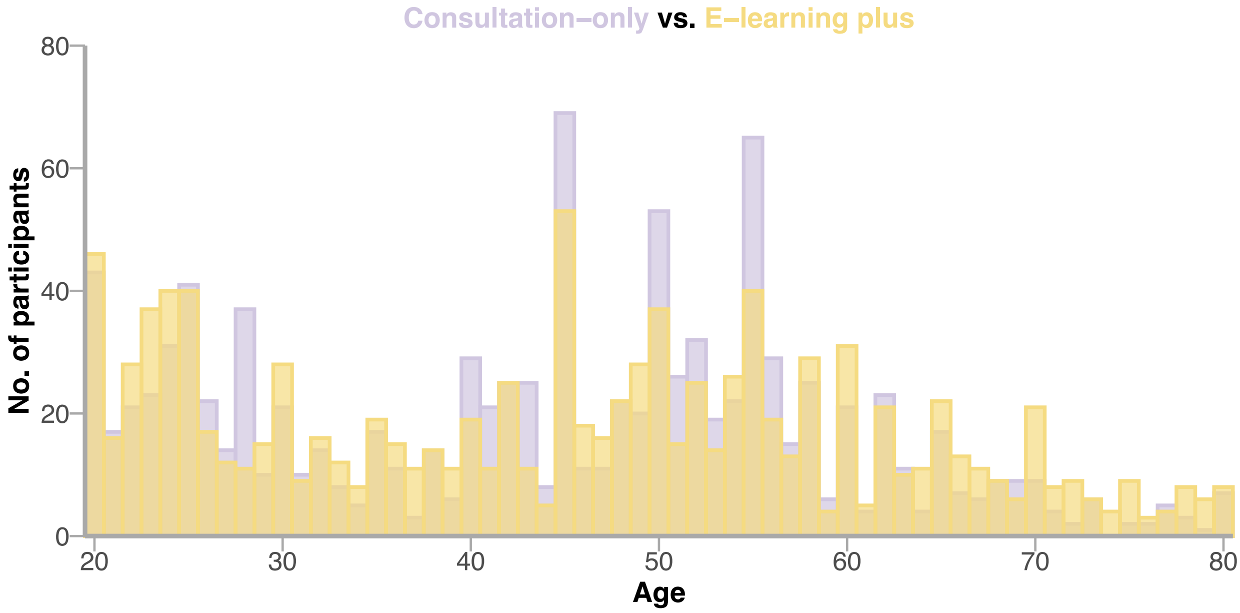

Supplementary Fig. 1 Age distribution of participants in the RCT.

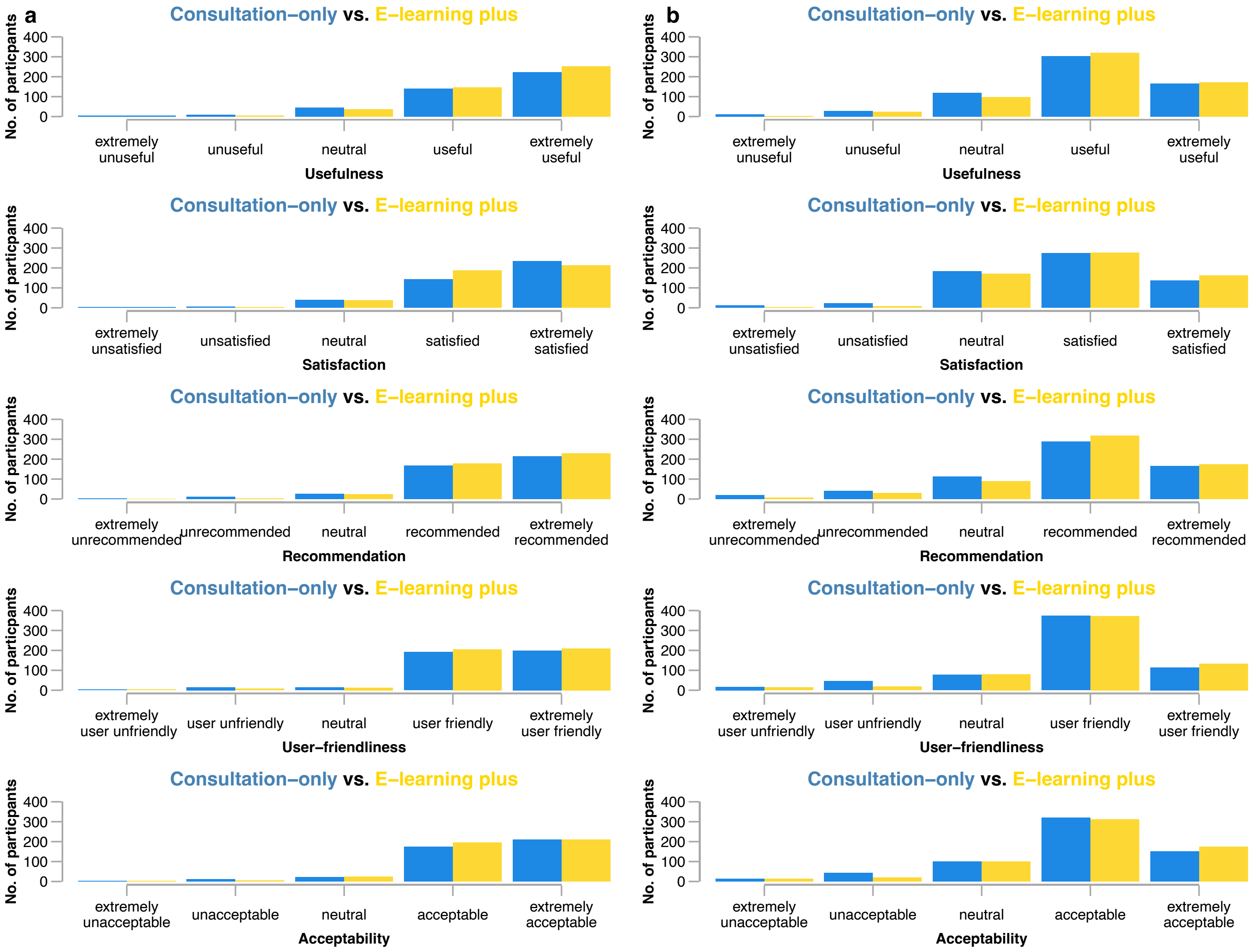

Supplementary Fig. 2 Distribution of patient feedback across five domains by rural and urban residents.

a, urban (n= 864 participants); **b**, rural (n= 1240 participants).

Demographic information

- **Gender**: Female
- **Age**: 66 years old
- **Height**: 166 cm
- **Weight**: 75 kg
- **Pregnancy Status**: No
- **Smoking**: No
- **Alcohol Consumption**: No

Clinical Presentation

- **Symptoms**:
  - Weight gain
  - Dyspnea (difficulty breathing)
  - Tachypnea (rapid breathing)
  - Wheezing
  - Cough
  - Productive cough (predominantly nocturnal)
  - Increased nocturnal awakenings
  - Dizziness or presyncope
  - Fatigue
  - Chest tightness
  - Lower limb edema (legs/ankles)
  - Exertional dyspnea requiring frequent rest breaks
  - Decreased appetite

Medical history

- **Family History**
  - Maternal history: Heart failure
  - Paternal uncle: Acute myocardial infarction
  - Paternal grandfather: Deceased due to stroke
  - Maternal grandmother: Deceased due to malignancy
- **Allergies:** No known allergies.

Referred Departments

- Pulmonology
- Cardiology

Supplementary Fig. 3 An illustrative example of a virtual patient profile.

Study protocol and statistical analysis plan

A randomized controlled trial on primary care using large language models and AI health literacy education

Protocol version: V1.0

Dates: January 17, 2025

Principal investigator:
Dr. Shasha Han, PhD
Assistant Professor, Chinese Academy of Medical Sciences & Peking Union Medical College

Sponsor:
Dr. Shasha Han, PhD
Assistant Professor, Chinese Academy of Medical Sciences & Peking Union Medical College

Funding: National Natural Science Foundation of China and Chinese Academy of Medical Sciences Innovation Fund. The sponsors and funders have no role in the study design, which includes the collection, management, analysis, and interpretation of data; the writing of the report; and the decision to submit the report for publication.

Table of Contents

Background and rationale 3
Objectives 4
Hypotheses 4
Methods 4
Trial design 4
Participants 4
Interventions 5
Outcomes 5
Recruitment plan 5
Randomization and blinding 5
Statistical analysis plan 6
Sample size 6
Statistical methods 6
Ethics 6
Good clinical practice 6
Protocol approval and amendments 7
Consent and assent 7
Confidentiality 7
Dissemination 7
References 7

Background and rationale

Primary care stands as the cornerstone of a high-functioning health system, yet it currently faces a pressing workforce and capacity crisis worldwide.[1,2] Additionally, the complexities of managing patients with multiple chronic and acute conditions require not only continuous care but also collaboration among multiple specialists. Addressing these challenges in primary care requires an innovative approach to healthcare delivery, with a strong emphasis on enhancing access and prioritizing patients in the community.

Large language models (LLMs) that generate humanlike text in response to user inquiries have shown the ability to solve complex patient cases, demonstrate humanlike clinical reasoning, gather patient histories, and communicate with empathetic tones in clinical settings.[3–6] The potential of LLMs in primary care is a source of growing enthusiasm.[7] LLMs could offer initial assessments, multilingual support, and individual-tailored health information and facilitate ongoing care and health management, especially in underserved communities and for those with limited mobility.[8] Besides, they can act as a proactive ally in promoting healthy behaviors and lifestyle changes, potentially preventing chronic diseases and alleviating pressure on healthcare systems.[9]

However, the successful integration of LLMs into community primary care is contingent upon individuals' proficiency in interacting with these technologies, a concept termed AI health literacy. Deficiencies in AI health literacy can lead to suboptimal interactions, wherein vague or overly generalized user input impedes the LLMs' capacity to generate clinically relevant responses. [10–13] Furthermore, inherent biases within training datasets and the potential for LLM hallucinations may produce contextually appropriate but factually inaccurate information, which demands critical evaluation and correction facilitated by robust AI literacy.[14–16] Compounding these challenges, the deployment of LLMs in community settings is further constrained by inadequate network infrastructure.[17,18] Reliable broadband internet connectivity cannot be universally assured[19], particularly in rural areas where it may be prohibitively expensive[20], and the absence of reliable neighborhood internet broadband access has been associated with diminished uptake of patient portals.[21]

To directly address the intertwined challenges of healthcare accessibility and AI health literacy in underserved communities, we introduce a novel co-design framework for developing and evaluating an LLM-powered primary care chatbot, termed P&P Care (Population Medicine & Public Health). Initially, we co-developed the foundational health consultation chatbot, leveraging GPT-4.0 (OpenAI; GPT-4o mini) for mobile accessibility. Subsequently, we co-designed targeted E-learning modules, comprising four key components—initiation control, termination control, duration control, and personalized content control—to enhance patients' AI health literacy. We then co-designed a pragmatic randomized controlled trial to evaluate the impact of these E-learning modules on the quality of health consultations and patient experiences with P&P Care, in comparison to a consultation-only control group. Our evaluation

focused on bidirectional human-LLM interaction, defining metrics beyond simple LLM responses to encompass patients' awareness of health needs and their perceptions of the communicative quality of P&P Care.

Through this work, we aim to demonstrate the significant value of co-designing LLM-powered primary care interventions to effectively bridge the AI health literacy gap in digital healthcare delivery [22], while addressing other implementation challenges within resource-constrained settings where such integrated solutions hold immense potential for impact.

Objectives

To investigate whether the E-learning modules improve awareness of health needs, enhance the quality of health consultations, and provide a better patient experience with the chatbot.

Hypotheses

Compared to the consultation-only group, the E-learning group will demonstrate enhanced awareness of health needs, improved quality of health consultations and better patient experiences with the P&P Care chatbot.

Methods

Trial design

This is a parallel randomized controlled trial involving community residents from across 11 provinces and autonomous regions in China, including both rural and urban communities. This geographically and culturally heterogeneous setting included Beijing, Chongqing, Gansu, Hubei, Shaanxi, Shandong, Shanxi, Sichuan, and the autonomous regions of Guangxi (Zhuang), Inner Mongolia (Mongolian), and Xinjiang (Uyghur). Participants will be randomly assigned to either the health consultation plus E-learning group or the health consultation-only group in a 1:1 ratio. We recorded the entire dialogue process and gathered participant feedback to evaluate the quality of the dialogue. Reporting adheres to the CONSORT-AI guidelines.[23]

Patients, caregivers, community health workers, and other stakeholders have been involved in the design of the interventions and the trial through the co-design process.

Participants

Inclusion Criteria: Participants must have a need for health consultation or express a willingness to engage in LLM-based health consultations. Additionally, participants must be aged between 20 and 80 years.

Exclusion Criteria: Individuals who present with psychological disorders or drug abuse and any other conditions that may compromise communicative interactions or the integrity of assessments.

Interventions

Participants are randomly assigned to one of two groups upon enrollment: the E-learning plus group or the consultation-only group. In the E-learning plus group, participants receive training on AI health literacy through an E-learning modules during the preparation phase, prior to engaging in health consultations. In contrast, participants in the consultation-only group undergo a controlled preparation where the E-learning modules are deactivated before they engage in health consultations. During the preparation phase, all participants are required to recall the symptoms and conditions of their recent and current illnesses. They then conduct health consultations with an AI counselor. Following the consultations, participants complete a post-consultation questionnaire to provide feedback on their experience.

Outcomes

The primary outcome measures comprise objective awareness of health needs. Secondary outcomes include attention, integrity, listenability, conciseness, and empathy. These outcomes serve as objective indicators of the consultation dialogue and were assessed by the same evaluation panel as in the P&P Care development. Panelists are blinded to participant allocation (E-learning versus consultation-only).

The secondary outcomes also include self-awareness of health needs, satisfaction, usefulness, user-friendliness, acceptability, and recommendation, which are retrieved from feedback questionnaires provided by the participants.

Recruitment plan

Our local co-designed teams, who live in the area and were proficient in the local dialect, will assist with subject recruitment and the trial. The co-designed team proactively contacts potential participants from the community who require LLM-based health consultations or are open to experiencing these consultations. For those who indicate interest, the teams will provide comprehensive descriptions of the study, emphasizing that it is exploratory and that any advice rendered by P&P Care serves solely as a reference and should not be utilized as a definitive basis for disease therapy. Participants will receive an informed consent form before enrollment and will have the opportunity to ask questions. After this process, potential participants who meet the established inclusion and exclusion criteria will be formally recruited.

Randomization and blinding

We use individual-level parallel randomization without stratification, utilizing a computer-generated random sequence for participant assignment to each experimental group. We will

implement allocation concealment to maintain the confidentiality of the random allocation and minimize bias. Throughout the intervention and analysis phases, the group information and operational materials will remain undisclosed to all researchers.

Statistical analysis plan

Sample size

Sample sizes were estimated based on the difference in awareness of health needs between E-learning plus and consultation-only groups. Using data from preliminary virtual experiments, we calculated that a sample size of 1200 participants would be required to achieve 80% power at a significance level of 0.05. To account for inflated literacy scores in virtual patients compared to real-world community residents, we plan to conduct an interim analysis after enrolling 500 participants to reestimate the sample size.

Statistical methods

To ensure objectivity, data collection and subsequent statistical analyses will be conducted by independent researchers. We will assess the normality of scale value distributions across all dimensions and use two-sample t-tests with unequal variances for intergroup comparisons where appropriate. For dimensions that exhibited significant skewness, we will employ non-parametric Mann-Whitney U tests. All statistical tests are two-tailed, with a significance threshold set at $P < 0.05$. Additionally, we apply the Benjamini-Hochberg adjustment for multiple testing corrections based on the total number of tests performed.

Subgroup analyses will be conducted based on age, community setting, sex, and whether the consultation involved multiple medical disciplines.

To establish real-world comparators for this randomized controlled trial, we plan to collect 100 routine clinical dialogue samples from primary care institutions in township and community settings. These samples will be used for quality outcome comparisons with the dialogues collected from the trial groups.

Ethics

Good clinical practice

This study will be conducted according to the highest ethical and scientific standards, adhering to Good Clinical Practice (GCP) guidelines (ICH E6 Guideline for Good Clinical Practice, 1 May 1996), the Declaration of Helsinki, and relevant local regulations.

Protocol approval and amendments

Before initiating a research study, the principal investigators will seek approval from the ethics committee for the research protocol, informed consent procedures, and any other written materials that will be provided to study participants. Throughout the study, particularly at critical junctures or when significant changes occur, the principal investigators will proactively update the ethics committee. The principal investigators will ensure that any amendments to the protocol or informed consent documents receive approval from the ethics committee prior to implementation, except in situations where immediate changes are essential to safeguard participant safety. In such instances, they will notify the ethics committee as promptly as possible.

Consent and assent

The investigators will ensure that study participants, or their legal representatives, fully understand the study and will provide written informed consent. Participants will receive ample time to consider their participation and will be instructed on how to sign the informed consent electronically. They will not be enrolled until they sign the informed consent. Throughout the study, participants will receive updated versions of the informed consent document and other written information as it becomes available. The investigator will retain the informed consent documents.

Confidentiality

The investigators will implement stringent data processing and anonymization procedures in the collection and use of data for this study. The data will be used exclusively for research purposes and strictly anonymized to minimize the risk of identifying individual participants. Additionally, throughout the study, the investigators will apply robust data privacy measures to further safeguard the confidentiality of personal information, ensuring that the integrity and security of the data remain uncompromised.

Dissemination

The principal investigators commit to communicating the results of this study and making them available for public disclosure. Public disclosure includes publishing a paper in a scientific journal, submitting an abstract for a poster or oral presentation at a scientific meeting, or other means of disclosure. No confidential information will be disclosed.

References

1. Jabbarpour Y, Jetty A, Byun H, Siddiqi A, Petterson S, Park J. The health of US primary care: 2024 scorecard report — No one can see you now. The Memorial Fund and The Physicians Foundation. Published online February 28, 2024.

2. Magon A, Caruso R. Addressing a potential crisis in the Italian National Health System. *The Lancet*. 2023;401(10384):1262-1263.

3. Savage T, Nayak A, Gallo R, Rangan E, Chen JH. Diagnostic reasoning prompts reveal the potential for large language model interpretability in medicine. *npj Digital Medicine 2024 7:1*. 2024;7(1):1-7.

4. Kanjee Z, Crowe B, Rodman A. Accuracy of a generative artificial intelligence model in a complex diagnostic challenge. *JAMA*. 2023;330(1):78-80.

5. Tu T, Palepu A, Schaekermann M, et al. Towards conversational diagnostic AI. Published online January 11, 2024.

6. Ayers JW, Poliak A, Dredze M, et al. Comparing physician and artificial intelligence chatbot responses to patient questions posted to a public social media forum. *JAMA Intern Med*. 2023;183(6):589-596.

7. Andrew A. Potential applications and implications of large language models in primary care. *Fam Med Community Health*. 2024;12(Suppl 1):e002602.

8. Wang X, Sanders HM, Liu Y, et al. ChatGPT: promise and challenges for deployment in low- and middle-income countries. *Lancet Reg Health West Pac*. 2023;41:100905.

9. Aggarwal A, Tam CC, Wu D, Li X, Qiao S. Artificial intelligence–based chatbots for promoting health behavioral changes: Systematic review. *J Med Internet Res 2023;25:e40789 https://www.jmir.org/2023/1/e40789*. 2023;25(1):e40789.

10. Meyer JG, Urbanowicz RJ, Martin PCN, et al. ChatGPT and large language models in academia: opportunities and challenges. *BioData Min*. 2023;16(1):1-11.

11. Tie J, Yao B, Li T, Ahmed SI, Wang D, Zhou S. LLMs are imperfect, then what? An empirical study on LLM failures in software engineering. Published online November 14, 2024.

12. Binz M, Schulz E. Using cognitive psychology to understand GPT-3. *Proc Natl Acad Sci U S A*. 2023;120(6):e2218523120.

13. Reynolds L, McDonell K. Prompt Programming for Large Language Models: Beyond the Few-Shot Paradigm. *Conference on Human Factors in Computing Systems - Proceedings*. Published online February 15, 2021.

14. Sallam M. ChatGPT utility in healthcare education, research, and practice: Systematic review on the promising perspectives and valid concerns. *Healthcare (Switzerland)*. 2023;11(6).

15. Shaikh O, Zhang H, Held WB, Bernstein M, Yang D. On second thought, let's not think step by step! Bias and toxicity in zero-shot reasoning. *Annual Meeting of the Association for Computational Linguistics*. Published online 2022.

16. Gilson A, Safranek CW, Huang T, et al. How does ChatGPT perform on the United States Medical Licensing Examination (USMLE)? The implications of large language models for medical education and knowledge assessment. *JMIR Med Educ*. 2023;9.

17. Challenges in digital medicine applications in under-resourced settings. *Nature Communications 2022 13:1*. 2022;13(1):1-5.

18. Yip W, Fu H, Jian W, et al. Universal health coverage in China part 2: addressing challenges and recommendations. *Lancet Public Health*. 2023;8(12):e1035-e1042.

19. China Internet Network Information Center. The 54 th statistical report on China's internet development. Published August 2024. Accessed January 11, 2025. https://www.cnnic.com.cn/IDR/ReportDownloads/202411/P020241101318428715781.pdf

20. Liu C, Wang L. Who is left behind? Exploring the characteristics of China's broadband non-adopting families. *Telecomm Policy*. 2021;45(9):102187.

21. Perzynski AT, Roach MJ, Shick S, et al. Patient portals and broadband internet inequality. *J Am Med Inform Assoc*. 2017;24(5):927.

22. Rajan D, Rouleau K, Winkelmann J, Jakab M, Kringos D, Khalid F. *Implementing the Primary Health Care Approach Global Report on Primary Health Care*.; 2024. Accessed March 24, 2025. https://iris.who.int/

23. Liu X, Cruz Rivera S, Moher D, et al. Reporting guidelines for clinical trial reports for interventions involving artificial intelligence: the CONSORT-AI extension. *Nat Med*. 2020;26(9):1364-1374.

研究方案和数据分析计划

基于大型语言模型的健康咨询和健康素养教育随机试验研究

方案版本：V1.0

版本日期：2025 年 1 月 17 日

项目负责人：韩莎莎，中国医学科学院北京协和医学院，群医学及公共卫生学院

项目资助情况：国自然科学基金和中国医学科学院创新工程基金

一、研究背景和意义

基层医疗作为现代医疗卫生体系的核心基础，当前正面临全球性的医疗人力资源短缺和服务能力不足的双重危机。[1,2] 基层医疗机构通常需要同时应对多种慢性病和急性病共患患者的诊疗管理，不仅要提供持续性的医疗照护，更需要建立多学科协同诊疗机制。在此背景下，创新基层医疗服务模式，提升社区医疗服务的可及性，构建以患者为中心的新型服务体系，已成为当务之急。

近年来，大型语言模型（Large Language Models, LLMs）在医疗健康领域展现出了一定的应用潜力。LLMs 不仅能够生成专业医疗知识回复，还具备解决复杂临床病例、模拟专业医疗推理、系统采集病史数据，以及实现共情沟通等多项能力。[3–6] 在初级卫生保健领域，LLMs 工具有望提供包括初步诊断评估、疾病预防、个性化健康指导等在内的多元化服务，以支持持续性全面健康管理，提升医疗资源匮乏地区和行动不便人群的高质量医疗资源可及性。[7, 8] 此外，LLMs 还能作为主动健康管理工具，通过促进健康行为改变和生活方式干预，在慢性病共病管理方面发挥重要作用，以缓解初级医疗系统人力资源缺的压力。[9]

然而，LLMs 在社区初级保健中的实际应用仍面临多重挑战：首先，用户必须具备足够的 AI 健康素养（即，个体运用 AI 技术的能力），才能有效使用这些工具。研究表明，AI 健康素养

不足会导致人机交互效率低下，用户输入的模糊性或泛化性会显著影响 LLMs 生成临床相关应答的质量[10–13]；其次，训练数据的固有偏差和 LLMs 潜在的幻觉问题，会导致 LLMs 生成看似合理但实际错误的回复，或对 AI 健康素养不足的用户产生误导。[14–16] 此外，社区网络基础设施的不完善也严重制约了 LLMs 的应用效果，特别是在宽带网络覆盖不足且费用高昂的农村地区，这一问题更为突出[17–21]。

针对上述挑战，本项目创新性地提出一项协同设计框架，且基于此框架开发并评估基于 LLMs 的初级保健聊天机器人系统 P&P Care（Population Medicine & Public Health）。研究将分三个阶段实施：1）基于 GPT-4.0（OpenAI GPT-4o mini）平台开发移动端基础健康咨询聊天机器人；2）设计包含启动控制、终止控制、时长控制和个性化内容控制四大模块的电子学习模块，专项提升用户 AI 健康素养；3）通过实用型随机对照试验，系统评估电子学习模块对咨询质量和使用体验的提升效果。项目特别关注人机双向交互质量，重点考察用户健康需求认知提升和咨询对话质量改善等协同设计过程中产生的测量指标。

本项目的实施将有力验证，在资源匮乏的环境中，协同设计的 LLMs 医疗干预解决方案，对于有效弥合数字医疗服务中人工智能健康素养鸿沟[22]，以及解决其他落地实施挑战具有重大价值，在提升当地基层医疗服务质量和效率方面具有很大潜力。

二、研究目的

本研究目的是探究电子学习模块是否能够提升社区居民对健康需求的认知水平、提高健康咨询服务对话质量、提升聊天机器人的使用体验。

三、研究假设

与未接受个性化学习的参与者相比，接受了个性化学习的参与者在 AI 健康咨询对话中展现出更高的健康需求认知水平，对话质量和咨询体验也更好。

四、研究方法

1. 研究设计

本研究采用平行式随机对照试验设计。研究对象是北京、重庆、甘肃、湖北、陕西、山东、山西、四川、广西、内蒙古、新疆等地的社区和乡镇居民。参与者将被 1:1 随机分配到“个性

化学习+AI 健康咨询”组和 “仅 AI 健康咨询”组。 完整的对话过程和参与者的反馈将被记录下来，用以判断对话的质量。将按照 CONSORT-AI 指南规范[23]，对研究进行报告。

患者、家属、社区卫生工作人员及其他相关利益人通过协同设计流程全程参与了干预方案及试验设计。

2. 研究参与者

2.1 研究纳入标准

有健康咨询需求或愿意进行 AI 体验健康咨询；年龄 20-80 岁。

2.2 研究排除标准

有心理障碍、药物滥用或其他可能影响沟通交流或评估完整性的个体。

3. 研究流程

参与者进入 AI 健康咨询体验项目即被随机分配到两个实验组。这项实验是双盲的，两个组实验参与者均会被告知将进行 AI 健康咨询准备，区别在于“个性化学习+AI 健康咨询”组的参与者在准备阶段会得到个性化学习培训，而“仅 AI 健康咨询”组不会得到个性化学习培训。在两组准备阶段，系统均将收集参与者的基本信息情况，指导参与者回忆本人最近的医学事件。在准备阶段结束之后，所有参与者均进入 AI 健康咨询程序，与 AI 健康咨询助手进行对话咨询。 完成咨询后，所有参与者均进入问卷测评环节进行体验反馈。

4. 主要测量指标或结局指标的选择和确认

主要结局指标为客观健康需求意识（Objective awareness of health needs），由第三方专家评审小组根据对话内容评判。专家评审小组成员对受试者分组情况采取盲法设计。

次要结局指标包括关注度（Attention）、友好度（Empathy）、完整性（Intergrity）、简洁性（Conciseness）、倾听性（Listenability），由第三方专家评审小组根据对话内容评判。专家评审小组成员对受试者分组情况采取盲法设计。

次要结局指标还包括主观健康需求意识（Self-awareness of health needs）、满意度（Satisifaction）、有用性（Usefulness）、用户友好性（User-friendliness）、接受度

（Acceptability）、推荐度（Recommendation），由患者进行在线问卷问答获得。

5. 受试者招募

由本地人员共同组建的团队（熟悉当地方言并长期居住在该地区）协助开展受试者招募工作。研究团队将主动联络在生活社区中有 AI 健康咨询需求或愿意尝试 AI 健康咨询体验的潜在受试者。对于表达参与兴趣的受试者，研究团队将提供详尽的研究说明，确保他们明确这是一项探索性质的试验，且 AI 咨询提供的建议性结果仅供参考，不可作为疾病治疗的直接依据。受试者将收到一份知情同意书，并有机会提出疑问。随后，符合纳入及排除标准的潜在受试者将被正式招募入组。

6. 随机化分组和盲法

本实验采用面向个体平行随机设计，无分层。使用计算机软件生成一个随机序列。参与者将根据该随机序列被分配到每个实验组。本实验将实施分配隐藏策略，以确保随机分配的保密性和减少偏倚。分组信息及组别操作材料在干预实施前不会对任何参与者或项目实施人员透露。在干预实施阶段，参与者及项目实施人员不会被告知其分组情况。

在本研究中，将对参与者、结局评估者和数据分析者实施盲法，以确保研究的客观性和结果的有效性。

五、数据分析计划

1. 样本量的计算和推理

本实验依据前期大语言模型在仿真实验的表现确立人群样本，在 0.05 显著性水平（β = 0.2）下达到 80%的功效。拟定 1200，两个实验组人数比例为 1：1。考虑到仿真实验中虚拟患者的健康素养水平可能普遍高于真实世界社区普通居民，我们计划在招募 500 名受试者后开展中期样本量再评估。

2. 数据分析

采取数据收集与后续分析人员分离的原则。数据分析人员将对十个维度的尺度值进行评估，以验证它们是否接近正态分布。在对比结局的尺度值时，将运用双样本 t 检验，并考虑不等方差的情况。针对数据分布高度偏斜的情形，将采用非参数 Mann–Whitney U 检验。所有统计

检验均设定为双尾，且显著性水平确定为 $P < 0.05$。此外，为进行多重测试校正，将根据所执行的检验总数应用 Benjamini–Hochberg 方法。

根据年龄、社区环境、性别和是否为多科室咨询进行亚组分析。

为建立本次随机对照试验的现实世界评估对照，我们拟从乡镇和社区基层医疗机构采集 100 份常规诊疗咨询对话样本，将其与受试者的咨询对话进行质量比较。

六、道德伦理

1. 临床试验规范

本研究将遵循最高伦理与科学标准，严格执行 《药物临床试验质量管理规范》(ICH E6, 1996 年 5 月 1 日版)、《赫尔辛基宣言》及相关相关法规要求。

2. 伦理委员会

在研究开始之前，研究者应获得伦理委员会对研究方案、知情同意书以及需要提供给研究参与者的其它书面信息的批准。在研究中止和/或完成时，研究者必须书面通知伦理委员会；研究者必须及时向伦理委员会报告所有研究工作中发生的变化（如方案和/或知情同意书的修订），并且在未获得伦理委员会批准之前不得执行这些变动，除非是为了消除对研究参与者明显且直接的风险而做出的变更，在发生这类情况时，将通知伦理委员会。

2. 参与者信息及知情同意

研究者必须向研究参与者或其监护人提供易于理解的并且经伦理委员会批准的知情同意书，并给与研究参与者或其监护人充分的时间考虑本项研究，在获得研究参与者签署的书面知情同意书之前，研究参与者不得入组。在研究参与者参与期间，将向研究参与者提供所有更新版本的知情同意书以及书面信息。知情同意书应作为临床研究的重要文档保留备查。

七、保密及数据安全

本试验数据收集中，数据将进行脱敏、去标识化处理。数据仅用于课题研究，且在研究过程中采用严格保密措施，以尽可能降低识别主体身份的风险，避免对数据信息主体产生不必要的风险。

八、研究结果的发布方式

本研究主要研究者承诺将及时公布研究结果并向公众公开。公开方式包括在科学期刊发表学术论文，向学术会议提交摘要，和其他适宜的公开途径。所有披露内容均不包含任何保密信息。

九、参考文献

1. Jabbarpour Y, Jetty A, Byun H, Siddiqi A, Petterson S, Park J. The health of US primary care: 2024 scorecard report — No one can see you now. The Memorial Fund and The Physicians Foundation. Published online February 28, 2024.
2. Magon A, Caruso R. Addressing a potential crisis in the Italian National Health System. The Lancet. 2023;401(10384):1262-1263.
3. Savage T, Nayak A, Gallo R, Rangan E, Chen JH. Diagnostic reasoning prompts reveal the potential for large language model interpretability in medicine. npj Digital Medicine 2024 7:1. 2024;7(1):1-7.
4. Kanjee Z, Crowe B, Rodman A. Accuracy of a generative artificial intelligence model in a complex diagnostic challenge. JAMA. 2023;330(1):78-80.
5. Tu T, Palepu A, Schaekermann M, et al. Towards conversational diagnostic AI. Published online January 11, 2024.
6. Ayers JW, Poliak A, Dredze M, et al. Comparing physician and artificial intelligence chatbot responses to patient questions posted to a public social media forum. JAMA Intern Med. 2023;183(6):589-596.
7. Andrew A. Potential applications and implications of large language models in primary care. Fam Med Community Health. 2024;12(Suppl 1):e002602.
8. Wang X, Sanders HM, Liu Y, et al. ChatGPT: promise and challenges for deployment in low- and middle-income countries. Lancet Reg Health West Pac. 2023;41:100905.
9. Aggarwal A, Tam CC, Wu D, Li X, Qiao S. Artificial intelligence–based chatbots for promoting health behavioral changes: Systematic review. J Med Internet Res 2023;25:e40789 https://www.jmir.org/2023/1/e40789. 2023;25(1):e40789.
10. Meyer JG, Urbanowicz RJ, Martin PCN, et al. ChatGPT and large language models in academia: opportunities and challenges. BioData Min. 2023;16(1):1-11.
11. Tie J, Yao B, Li T, Ahmed SI, Wang D, Zhou S. LLMs are imperfect, then what? An empirical study on LLM failures in software engineering. Published online November 14, 2024.

12. Binz M, Schulz E. Using cognitive psychology to understand GPT-3. Proc Natl Acad Sci U S A. 2023;120(6):e2218523120.

13. Reynolds L, McDonell K. Prompt Programming for Large Language Models: Beyond the Few-Shot Paradigm. Conference on Human Factors in Computing Systems - Proceedings. Published online February 15, 2021.

14. Sallam M. ChatGPT utility in healthcare education, research, and practice: Systematic review on the promising perspectives and valid concerns. Healthcare (Switzerland). 2023;11(6).

15. Shaikh O, Zhang H, Held WB, Bernstein M, Yang D. On second thought, let's not think step by step! Bias and toxicity in zero-shot reasoning. Annual Meeting of the Association for Computational Linguistics. Published online 2022.

16. Gilson A, Safranek CW, Huang T, et al. How does ChatGPT perform on the United States Medical Licensing Examination (USMLE)? The implications of large language models for medical education and knowledge assessment. JMIR Med Educ. 2023;9.

17. Challenges in digital medicine applications in under-resourced settings. Nature Communications 2022 13:1. 2022;13(1):1-5.

18. Yip W, Fu H, Jian W, et al. Universal health coverage in China part 2: addressing challenges and recommendations. Lancet Public Health. 2023;8(12):e1035-e1042.

19. China Internet Network Information Center. The 54th statistical report on China's internet development. Published August 2024. Accessed January 11, 2025.

https://www.cnnic.com.cn/IDR/ReportDownloads/202411/P020241101318428715781.pdf

20. Liu C, Wang L. Who is left behind? Exploring the characteristics of China's broadband non-adopting families. Telecomm Policy. 2021;45(9):102187.

21. Perzynski AT, Roach MJ, Shick S, et al. Patient portals and broadband internet inequality. J Am Med Inform Assoc. 2017;24(5):927.

22. Rajan D, Rouleau K, Winkelmann J, Jakab M, Kringos D, Khalid F. Implementing the Primary Health Care Approach Global Report on Primary Health Care.; 2024. Accessed March 24, 2025. https://iris.who.int/

23. Liu X, Cruz Rivera S, Moher D, et al. Reporting guidelines for clinical trial reports for interventions involving artificial intelligence: the CONSORT-AI extension. Nat Med. 2020;26(9):1364-1374.
