# Supplementary material for: A community-codesigned LLM-powered chatbot for primary care: a randomized controlled trial
Source: Nat Health. 2026 Jan 19;1(2):238–50. doi: 10.1038/s44360-025-00021-w (PMC12880911; doi:10.1038/s44360-025-00021-w)
Supplement: Supplementary file 2 — Reporting Summary [file 44360_2025_21_MOESM2_ESM.pdf]

Corresponding author(s): Shasha Han
Last updated by author(s): Oct 15, 2025

Reporting Summary

Nature Portfolio wishes to improve the reproducibility of the work that we publish. This form provides structure for consistency and transparency in reporting. For further information on Nature Portfolio policies, see our Editorial Policies and the Editorial Policy Checklist.

Statistics

For all statistical analyses, confirm that the following items are present in the figure legend, table legend, main text, or Methods section.

n/a	Confirmed	
☐	☒	The exact sample size (n) for each experimental group/condition, given as a discrete number and unit of measurement
☐	☒	A statement on whether measurements were taken from distinct samples or whether the same sample was measured repeatedly
☐	☒	The statistical test(s) used AND whether they are one- or two-sided<br>*Only common tests should be described solely by name; describe more complex techniques in the Methods section.*
☐	☒	A description of all covariates tested
☐	☒	A description of any assumptions or corrections, such as tests of normality and adjustment for multiple comparisons
☐	☒	A full description of the statistical parameters including central tendency (e.g. means) or other basic estimates (e.g. regression coefficient) AND variation (e.g. standard deviation) or associated estimates of uncertainty (e.g. confidence intervals)
☐	☒	For null hypothesis testing, the test statistic (e.g. F, t, r) with confidence intervals, effect sizes, degrees of freedom and P value noted<br>*Give P values as exact values whenever suitable.*
☒	☐	For Bayesian analysis, information on the choice of priors and Markov chain Monte Carlo settings
☒	☐	For hierarchical and complex designs, identification of the appropriate level for tests and full reporting of outcomes
☐	☒	Estimates of effect sizes (e.g. Cohen's d, Pearson's r), indicating how they were calculated

Our web collection on statistics for biologists contains articles on many of the points above.

Software and code

Policy information about availability of computer code

Data collection	Our co-designed GPT-4-powered P&P Care (OpenAI; GPT-4o mini) chatbot was used to collect text data and the platform was used to collect the survey data.
Data analysis	R 4.3.0 was used to perform the statistical analyses and present the results. Comparative statistical analyses were detailed in the paper. Code for data visualization can be found at the following link (https://github.com/ShashaHan-collab/PaP-CoummunityRCT).

For manuscripts utilizing custom algorithms or software that are central to the research but not yet described in published literature, software must be made available to editors and reviewers. We strongly encourage code deposition in a community repository (e.g. GitHub). See the Nature Portfolio guidelines for submitting code & software for further information.

Data

Policy information about availability of data

All manuscripts must include a data availability statement. This statement should provide the following information, where applicable:

- Accession codes, unique identifiers, or web links for publicly available datasets
- A description of any restrictions on data availability
- For clinical datasets or third party data, please ensure that the statement adheres to our policy

The study protocol is provided in the Supplementary Information. Source data are provided in Tables and Extended Data Tables and can be accessed via the code repository (https://github.com/ShashaHan-collab/ PaP-CoummunityRCT). Raw conversation data are not publicly available due to the need to protect participant

privacy, in accordance with the ethical approval for this study. Anonymized, non-dialogue individual-level data underlying the results can be requested by qualified researchers for academic use. Requests should include a research proposal, statistical analysis plan, and justification for data use, and can be submitted via email to S.H. (hanshasha@pumc.edu.cn). All requests will be reviewed by the Chinese Academy of Medical Sciences & Peking Union Medical College. Applicants will receive an initial response within two months, and approved requests will be granted access via a secure platform after execution of a data access agreement.

Research involving human participants, their data, or biological material

Policy information about studies with human participants or human data. See also policy information about sex, gender (identity/presentation), and sexual orientation and race, ethnicity and racism.

Reporting on sex and gender

Our trial analysis included 2,113 participants (1060 with E-learning plus and 1053 with consultation-only), with a mean age of 44.5 (SD 15.7) and 1,052 (49.8%) being females.
Subgroup analyses were performed based on age, community setting, sex, and ICD-10 domains.
Female participants demonstrated comparable improvements in awareness to male participants but exhibited superior gains in the remaining five metrics. Moreover, participants requiring multiple ICD-10 domain consultations experienced greater improvements compared to those seeking single domain consultations (Extended Data Table 3).

Reporting on race, ethnicity, or other socially relevant groupings

1077 (51.0%) needing consulting more than one medical discipline.1,249 (59.1%) of participants were from rural areas.
Detailed demographics of the participants are provided in Fig. 2b.
Although randomization balanced baseline characteristics, we acknowledge potential influences on internal validity. The E-learning group's longer preparation time may have heightened interaction burden, potentially influencing self-reported satisfaction. However, this performance bias risk was mitigated by blinded assessment of objective endpoints.

Population characteristics

See above and Figure 2b

Recruitment

Our local co-designed teams, who lived in the area and were proficient in the local dialect, assisted with subject recruitment and the trial. The co-designed team proactively contacted potential participants from the community who require LLM-based health consultations or are open to experiencing these consultations. For those who indicate interest, the teams provided comprehensive descriptions of the study, emphasizing that it is exploratory and that any advice rendered by P&P Care serves solely as a reference and should not be utilized as a definitive basis for disease therapy. Participants received an informed consent form before enrollment and had the opportunity to ask questions. After this process, potential participants who met the established inclusion and exclusion criteria were formally recruited.

Ethics oversight

The study protocol received approval from the Ethics Review Committee of the Chinese Academy of Medical Sciences & Peking Union Medical Colleges and was prospectively registered with the Chinese Clinical Trial Registry (identifier: ChiCTR2500098101). All participants provided informed consent in accordance with the Declaration of Helsinki, with explicit disclosure that this is an exploratory experiment and that the chatbot health advice given during consultations should not be used for disease management without clinician oversight. Stringent data protection protocols were implemented in this study, ensuring that all data were anonymized and encrypted for privacy protection.

Note that full information on the approval of the study protocol must also be provided in the manuscript.

Field-specific reporting

Please select the one below that is the best fit for your research. If you are not sure, read the appropriate sections before making your selection.

☒ Life sciences ☐ Behavioural & social sciences ☐ Ecological, evolutionary & environmental sciences

For a reference copy of the document with all sections, see nature.com/documents/nr-reporting-summary-flat.pdf

Life sciences study design

All studies must disclose on these points even when the disclosure is negative.

Sample size

Sample sizes were estimated based on the difference in awareness of health needs between E-learning plus and consultation-only groups. Using data from preliminary virtual experiments, we calculated that a sample size of 1200 participants would be required to achieve 80% power at a significance level of 0.05. To account for inflated literacy scores in virtual patients compared to real-world community residents, we conducted an interim analysis after enrolling 500 participants. This adjustment necessitated recalculating the differences, requiring a revised sample size of 2,000 participants to maintain statistical power.

Data exclusions

2,202 participants were randomly assigned to the E-learning plus group (n= 1,096) or the consultation-only group (n= 1,106) using sealed envelopes for a straightforward 1:1 allocation. Of these, 58 participants later chose to opt out for personal reasons, and 31 quit due to network disruptions.

Replication

Stratified analyses revealed that E-learning conferred disproportionately greater benefits to specific participant subgroups. Notably, older participants demonstrated a significantly higher increase in awareness following E-learning compared to younger participants. These findings suggest that E-learning effectively enhances health-related awareness and communication skills among older adults. Rural participants exhibited smaller improvements across all six evaluation metrics when compared to their urban counterparts. These findings indicate that E-learning yielded diminished gains in rural settings, potentially reflecting variations in engagement with E-learning.

Randomization	We used individual-level parallel randomization without stratification, utilizing a computer-generated random sequence for participant assignment to each experimental group.
Blinding	We implemented allocation concealment to maintain the confidentiality of the random allocation and minimize bias. Throughout the intervention and analysis phases, the group information and operational materials remained undisclosed to all researchers.

Reporting for specific materials, systems and methods

We require information from authors about some types of materials, experimental systems and methods used in many studies. Here, indicate whether each material, system or method listed is relevant to your study. If you are not sure if a list item applies to your research, read the appropriate section before selecting a response.

Materials & experimental systems

n/a	Involved in the study
☒	☐ Antibodies
☒	☐ Eukaryotic cell lines
☒	☐ Palaeontology and archaeology
☒	☐ Animals and other organisms
☐	☒ Clinical data
☒	☐ Dual use research of concern
☒	☐ Plants

Methods

n/a	Involved in the study
☒	☐ ChIP-seq
☒	☐ Flow cytometry
☒	☐ MRI-based neuroimaging

Clinical data

Policy information about clinical studies

All manuscripts should comply with the ICMJE guidelines for publication of clinical research and a completed CONSORT checklist must be included with all submissions.

Clinical trial registration	Chinese Clinical Trial Registry identifier: ChiCTR2500098101
Study protocol	The study protocol is provided with this Reporting Summary.
Data collection	The study was conducted across a wide range of 11 provinces and autonomous regions in China, specifically selected to ensure geographical and cultural heterogeneity. Data collection took place at the community level in both rural and urban settings.<br><br>The specific locations are detailed by region. Sites included Fengtai District and Miyun District in Beijing; Youyang County in Chongqing; Kongtong District in Gansu; Gongan County and Jiangxia District in Hubei; Fuping County and Yaozhou District in Shaanxi; Dongping County, Jinxiang County, and Pingdu County in Shandong; Yaodu District in Shanxi; Weiyuan County, Yantan District and Gongjing District in Sichuan; Changzhou District and Wanxiu District in Guangxi; Saihan District in Inner Mongolia; and Aksu District and Xinhe County in Xinjiang.<br><br>Participant recruitment for this trial was conducted from March 4th to March 30th, 2025. Based on standard protocols, the primary data collection have occurred concurrently with this recruitment period.
Outcomes	The primary outcome measures comprised objective awareness of health needs. Secondary outcomes included attention, integrity, listenability, conciseness, and empathy. These outcomes serve as objective indicators of the consultation dialogue and were assessed by the same evaluation panel as in the P&P Care development. Panelists were blinded to participant allocation (E-learning versus consultation-only). The secondary outcomes also include self-awareness of health needs, satisfaction, usefulness, accessibility, acceptability, and recommendation, which were retrieved from feedback questionnaires provided by the participants.

Plants

Seed stocks	*Report on the source of all seed stocks or other plant material used. If applicable, state the seed stock centre and catalogue number. If plant specimens were collected from the field, describe the collection location, date and sampling procedures.*
Novel plant genotypes	*Describe the methods by which all novel plant genotypes were produced. This includes those generated by transgenic approaches, gene editing, chemical/radiation-based mutagenesis and hybridization. For transgenic lines, describe the transformation method, the number of independent lines analyzed and the generation upon which experiments were performed. For gene-edited lines, describe the editor used, the endogenous sequence targeted for editing, the targeting guide RNA sequence (if applicable) and how the editor was applied.*
Authentication	*Describe any authentication procedures for each seed stock used or novel genotype generated. Describe any experiments used to assess the effect of a mutation and, where applicable, how potential secondary effects (e.g. second site T-DNA insertions, mosiacism, off-target gene editing) were examined.*
